# Incremental value of insulin resistance surrogate indices beyond the CHA_2_DS_2_-VASc score for predicting thromboembolic events in patients with chronic heart failure and atrial fibrillation

**DOI:** 10.3389/fendo.2026.1954348

**Published:** 2026-09-23

**Authors:** Yingli Xie, Yanhui Han, You Zhou, Huifeng Zhang

**Affiliations:** Department of Cardiology, The First Affiliated Hospital, and College of Clinical Medicine of Henan University of Science and Technology, Luoyang, Henan, China

**Keywords:** atrial fibrillation, CHA2DS2-VASc score, chronic heart failure, insulin resistance, thromboembolic events

## Abstract

**Background:**

Patients with chronic heart failure (CHF) and atrial fibrillation (AF) are at high risk of thromboembolic events. Although the CHA_2_DS_2_-VASc score is widely used for risk stratification, it may not fully capture metabolic dysfunction. Whether insulin resistance (IR) surrogate indices provide incremental information beyond the CHA_2_DS_2_-VASc score in patients with CHF and AF remains unclear.

**Methods:**

This retrospective cohort study included 4,474 patients with CHF and AF. IR surrogate indices were calculated, and thromboembolic events were defined as ischemic stroke or systemic embolism (IS/SE). Multivariable Cox regression models assessed the associations between these indices and thromboembolic risk. Their incremental value beyond the CHA_2_DS_2_-VASc score for 4-year IS/SE risk was evaluated using discrimination, reclassification, and clinical utility measures, with stratification by AF type.

**Results:**

During a median follow-up of 4.00 years, 349 IS/SE events occurred. After multivariable adjustment, the hazard ratios per 1-SD increase were 1.27 (95% confidence interval, 1.13–1.43) for the TyG index, 1.41 (1.28–1.56) for the TyG-BMI index, 1.27 (1.20–1.34) for METS-IR, and 1.13 (1.08–1.19) for the TG/HDL-C ratio (all P < 0.001). Risk increased progressively across tertiles of each index, and the associations remained consistent in competing-risk analyses. Adding METS-IR or the TyG-BMI index to the CHA_2_DS_2_-VASc score significantly increased the AUC from 0.637 to 0.682 and 0.673, respectively, and improved reclassification. Decision curve analysis showed greater net benefit for models incorporating IR surrogate indices, particularly METS-IR and the TyG-BMI index. The incremental predictive improvements appeared more evident in patients with non-paroxysmal AF.

**Conclusions:**

IR surrogate indices were independently associated with IS/SE in patients with CHF and AF. METS-IR and the TyG-BMI index provided the most consistent incremental information beyond the CHA_2_DS_2_-VASc score, with more evident improvements observed in patients with non-paroxysmal AF.

## Introduction

Chronic heart failure (CHF) and atrial fibrillation (AF) frequently coexist in clinical practice and jointly identify a population with substantial cardiovascular risk. Recent evidence has emphasized the close interaction between AF and heart failure, including shared risk factors, bidirectional pathophysiological mechanisms, and adverse prognostic implications (1). CHF is associated with an increased risk of stroke, and concomitant AF may further amplify thromboembolic vulnerability (2). The presence of AF also forms the basis for structured stroke prevention strategies in contemporary guidelines (3). Therefore, accurate identification of thromboembolic risk in patients with CHF and AF remains clinically important.

Current stroke prevention strategies in AF rely heavily on clinical risk assessment. The CHA_2_DS_2_-VASc score, which incorporates major clinical risk factors, remains a widely used and guideline-endorsed tool for thromboembolic risk assessment and anticoagulation decision-making in patients with AF (3). However, although this score is simple and widely used, its discriminatory performance for ischemic stroke has been reported to be modest (4). This limitation may be particularly relevant in patients with CHF and AF, in whom thromboembolic risk may also be influenced by metabolic dysfunction, endothelial injury, inflammatory activation, and prothrombotic pathways (2, 5). Accordingly, readily available markers reflecting residual metabolic risk may complement established clinical risk scores.

Insulin resistance (IR), a central feature of metabolic dysfunction, has been implicated in adverse cardiovascular and cerebrovascular outcomes. IR may contribute to ischemic stroke through vascular inflammation, oxidative stress, endothelial dysfunction, platelet activation, thrombosis, and atherosclerotic progression (5), and genetic evidence has supported causal associations of IR with coronary artery disease and ischemic stroke (6). In addition, IR may be involved in atrial remodeling and AF-related arrhythmogenesis (7). Because direct assessment of IR using the hyperinsulinemic-euglycemic clamp is impractical in routine clinical settings, several simple surrogate indices have been developed. Among them, the triglyceride-glucose (TyG) index, triglyceride-glucose body mass index (TyG-BMI), metabolic score for insulin resistance (METS-IR), and triglyceride to high-density lipoprotein cholesterol (TG/HDL-C) ratio have been used as simple surrogate markers of IR in cardiometabolic research (8–11). These indices are derived from routine lipid, glucose, and anthropometric measurements, do not require insulin testing, and are simple, low-cost, and readily available.

Despite their biological plausibility and clinical availability, the prognostic relevance of IR surrogate indices for thromboembolic events in patients with CHF and AF remains unclear. It is also uncertain whether these indices provide incremental predictive value beyond the CHA_2_DS_2_-VASc score. AF type may reflect differences in arrhythmia burden and underlying atrial substrate. Non-paroxysmal AF has been associated with greater thromboembolic risk than paroxysmal AF (12), while higher AF burden is linked to increased ischemic stroke risk (13). Moreover, IR may promote atrial structural and electrical remodeling through inflammatory, oxidative, and fibrotic pathways (7), suggesting that its prognostic relevance may differ across AF phenotypes. We therefore conducted AF-type-stratified analyses as a clinically driven exploratory assessment of potential heterogeneity. In this study, we aimed to evaluate the associations of multiple IR surrogate indices with thromboembolic events, their incremental value beyond the CHA_2_DS_2_-VASc score, and whether these findings differed between paroxysmal and non-paroxysmal AF (**Figure 1**).

**Figure 1:**
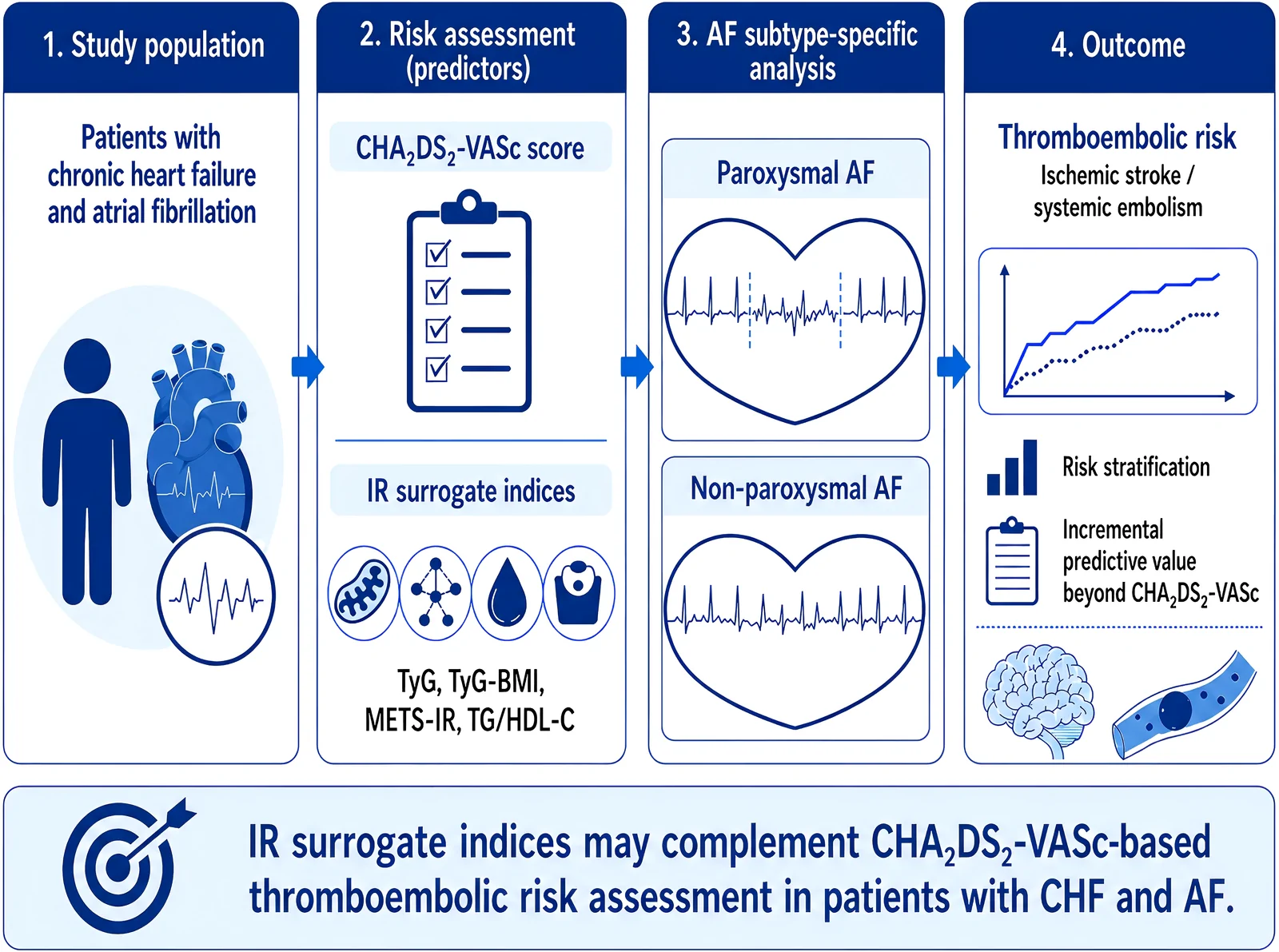
Analytical framework for evaluating IR surrogate indices in CHA_2_DS_2_-VASc-based IS/SE risk assessment among patients with CHF and AF. *IR*, insulin resistance; *IS/SE*, ischemic stroke/systemic embolism; *CHF*, chronic heart failure; *AF*, atrial fibrillation; *TyG*, triglyceride-glucose; *TyG-BMI*, triglyceride-glucose body mass index; *METS-IR*, metabolic score for insulin resistance; *TG/HDL-C*, triglyceride to high-density lipoprotein cholesterol ratio.

## Methods

### Study design and population

This retrospective study included patients hospitalized with CHF and AF at the First Affiliated Hospital of Henan University of Science and Technology and two medical centers of the Chinese PLA General Hospital between January 1, 2017, and January 31, 2023. Patients with AF in the presence of mechanical prosthetic valves or significant mitral stenosis were not included during cohort construction. CHF was diagnosed according to the 2021 ESC Guidelines for the diagnosis and treatment of acute and chronic heart failure (14). AF was diagnosed according to the 2024 ESC Guidelines for the management of atrial fibrillation (15). AF type was ascertained from the clinical documentation available at the index hospitalization. Paroxysmal AF was defined as AF that terminated spontaneously or with intervention within 7 days of onset; persistent, long-standing persistent, and permanent AF were collectively categorized as non-paroxysmal AF.

Patients were excluded if they met any of the following criteria: (1) age < 18 or > 90 years, or pregnancy; (2) connective tissue disease or malignant tumors; (3) severe hepatic or renal dysfunction; (4) missing data required to calculate IR surrogate indices, including fasting blood glucose (FBG), body mass index (BMI), triglycerides (TG), or high-density lipoprotein cholesterol (HDL-C); (5) died during hospitalization; and (6) were lost to follow-up. After applying these exclusion criteria, a total of 4,474 patients were included in the final analysis (**Figure 2**).

**Figure 2:**
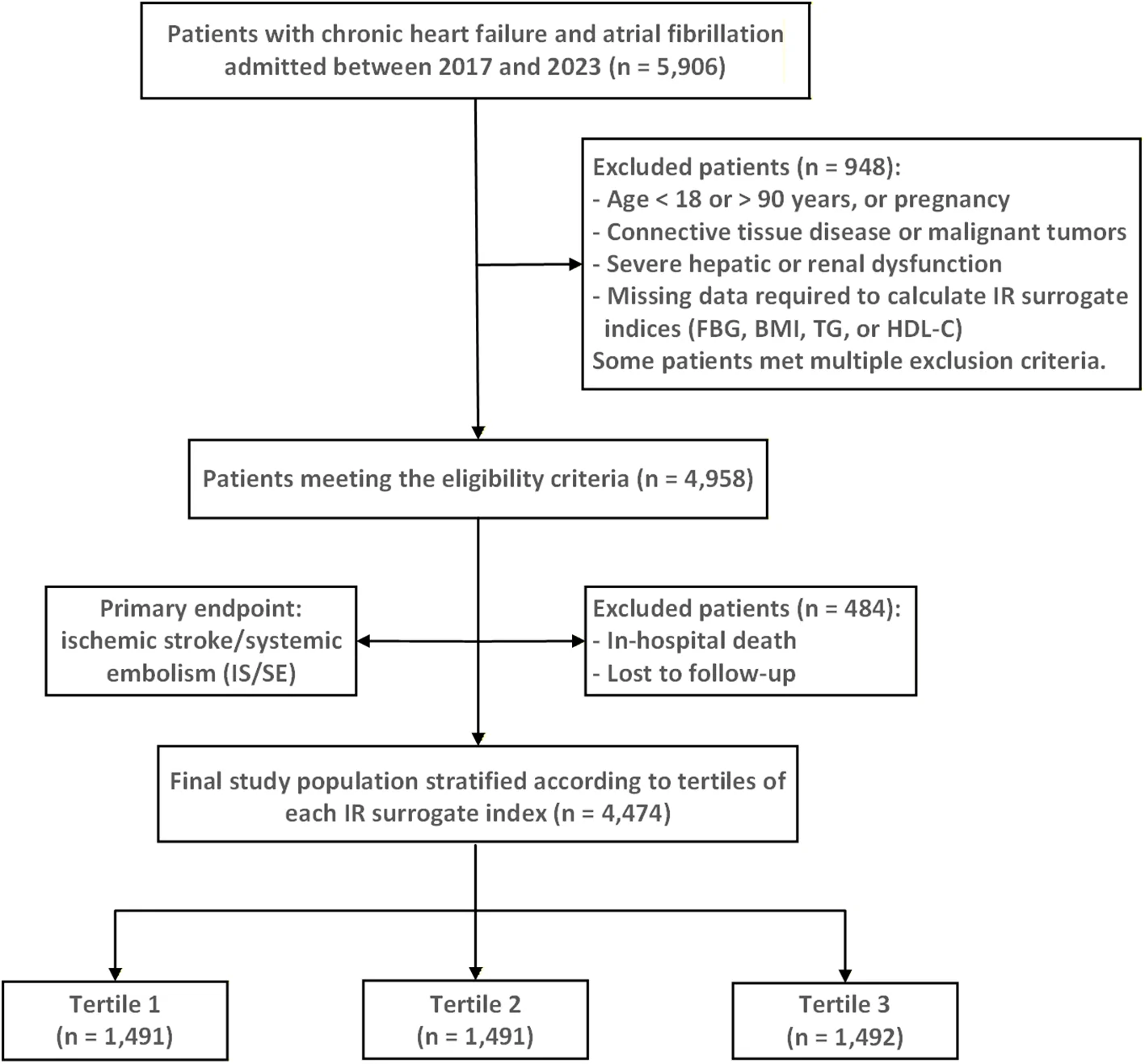
Flow diagram of study population selection. *IR*, insulin resistance; *FBG*, fasting blood glucose; *BMI*, body mass index; *TG*, triglyceride; *HDL-C*, high-density lipoprotein cholesterol; *IS/SE*, ischemic stroke/systemic embolism.

The IR surrogate indices evaluated in this study included the TyG index, TyG-BMI index, METS-IR, and TG/HDL-C ratio. These indices were analyzed both as standardized continuous variables and according to tertiles based on their distributions in the study population. AF-type-stratified analyses were undertaken as clinically motivated exploratory secondary analyses to examine potential heterogeneity in the associations and incremental predictive value of IR surrogate indices between paroxysmal and non-paroxysmal AF. These analyses were regarded as hypothesis-generating rather than confirmatory.

### Ethics statement

This study was conducted in accordance with the principles of the Declaration of Helsinki and was approved by the Ethics Committee of The First Affiliated Hospital of Henan University of Science and Technology (approval No. 2026-03-H0066). Owing to the retrospective nature of the study and the use of anonymized clinical data, the study was conducted under the institutional general consent framework or with a waiver of additional individual informed consent approved by the ethics committee, as appropriate. All patient-related information was anonymized before analysis to protect patient privacy.

### Data collection and definitions

Baseline demographic characteristics, comorbidities, laboratory measurements, echocardiographic parameters, and medication use were extracted from electronic medical record systems. Baseline medication use was determined from prescriptions at hospital discharge, and oral anticoagulant therapy included warfarin and direct oral anticoagulants. BMI was calculated as body weight in kilograms divided by the square of height in meters. Mean arterial pressure (MAP) was calculated as follows: (systolic blood pressure + 2 × diastolic blood pressure)/3. The CHA_2_DS_2_-VASc score was calculated according to established criteria (16).

The IR surrogate indices were calculated using previously reported formulas (8–11). The TyG index was calculated as ln [TG (mg/dL) × FBG (mg/dL)/2]. The TyG-BMI index was calculated as TyG index × BMI. METS-IR was calculated as (ln [2 × FBG (mg/dL) + TG (mg/dL)] × BMI)/ln [HDL-C (mg/dL)]. The TG/HDL-C ratio was calculated as TG divided by HDL-C.

### Follow-up and outcomes

The primary endpoint was the first occurrence of ischemic stroke or systemic embolism (IS/SE). Follow-up information was obtained through telephone interviews and review of electronic medical records. The median follow-up duration was 4.0 years (IQR, 2.5–6.1 years).

### Statistical analysis

Continuous variables were expressed as mean ± standard deviation (SD) or median with IQR, according to data distribution, and were compared using Student’s t test or one-way analysis of variance for normally distributed variables and the Mann–Whitney U test or Kruskal–Wallis test for non-normally distributed variables, as appropriate. Categorical variables were presented as frequencies and percentages, and comparisons were performed using the chi-square test or Fisher’s exact test, as appropriate. Missing data for baseline covariates were handled using multiple imputation by chained equations. To facilitate the assessment of potential confounding, baseline characteristics were additionally compared across tertiles of each IR surrogate index using the aforementioned statistical methods.

The four IR surrogate indices were analyzed both as continuous variables per 1-SD increase and as categorical variables according to tertiles, with the lowest tertile used as the reference group. Kaplan–Meier curves were generated to estimate IS/SE-free survival according to tertiles of each IR surrogate index, and differences between groups were assessed using the log-rank test.

Univariable and multivariable Cox proportional hazards regression models were used to evaluate the associations between IR surrogate indices and the risk of IS/SE. Covariates were selected based on their clinical relevance and known or plausible relationships with the IR surrogate indices and IS/SE, with additional consideration of the univariable Cox regression results (**Supplementary Table S1**). Model 1 was adjusted for age, sex, and smoking status. Model 2 was further adjusted for Model 1 covariates plus variables related to vital signs, cardiac function, laboratory measurements, comorbidities, ischemic etiology, and medication use. Components of each IR surrogate index were excluded from the corresponding Model 2 to avoid potential collinearity. Tests for linear trend across tertiles were performed by assigning the median value of each tertile and modeling this variable as continuous. Additional analyses included diabetes-stratified Cox models and Fine–Gray models treating death before IS/SE as a competing event, using the same model specifications.

Restricted cubic spline (RCS) analysis with three knots was performed to explore the dose–response associations between each IR surrogate index and the risk of IS/SE. Associations were subsequently estimated separately in patients with paroxysmal and non-paroxysmal AF. Potential effect modification by AF type was formally evaluated by including multiplicative interaction terms between AF type and each standardized IR surrogate index in the fully adjusted Cox models. Given the exploratory nature of these analyses, a *P* for interaction < 0.10 was considered suggestive, rather than definitive, evidence of potential heterogeneity.

To assess the incremental predictive value of IR surrogate indices beyond the CHA_2_DS_2_-VASc score, time-dependent receiver operating characteristic (ROC) curve analysis was performed for 4-year IS/SE risk, and area under the curve (AUC) values were calculated and compared. Improvements in risk discrimination and reclassification were further evaluated using continuous net reclassification improvement (NRI) and integrated discrimination improvement (IDI) (17). Decision curve analysis (DCA) was performed to evaluate the clinical net benefit of adding each IR surrogate index to the CHA_2_DS_2_-VASc score (18).

All statistical analyses were performed using R software version 4.2.1 (R Foundation for Statistical Computing, Vienna, Austria). A two-tailed *P* value < 0.05 was considered statistically significant, except for interaction analyses as specified above.

## Results

### Characteristics of the participants

A total of 4,474 patients with CHF and AF were included in the final analysis, including 1,251 with paroxysmal AF and 3,223 with non-paroxysmal AF. The baseline characteristics stratified by AF type are shown in **Table 1**. Compared with patients with paroxysmal AF, those with non-paroxysmal AF were slightly older, had a higher CHA_2_DS_2_-VASc score, and showed higher proportions of hypertension, diabetes, prior stroke, peripheral arterial disease, and ischemic etiology (all *P* < 0.05). In contrast, sex distribution, smoking status, BMI, MAP, heart rate, New York Heart Association class, and chronic kidney disease were comparable between the two groups.

**Table 1:** Baseline characteristics of patients with CHF and AF stratified by AF type.

Variables	Paroxysmal AF (n=1251)	Non-paroxysmal AF (n=3223)	*P* value
Demographics			
Age (years)	65.0 (55.0-74.0)	66.0 (57.0-76.0)	<0.001
Male (%)	750 (59.95%)	1935 (60.04%)	0.958
Current/ex-Smoker (%)	386 (30.86%)	1031 (31.99%)	0.465
Medical measurements			
BMI (kg/m^2^)	24.8 (22.2-27.6)	24.9 (22.7-27.4)	0.090
MAP (mmHg)	94.8 ± 13.0	95.3 ± 13.5	0.270
HR (bpm)	78.0 (68.0-89.0)	78.0 (67.0-88.0)	0.432
NYHA classification (%)			0.386
I-II	516 (41.25%)	1260 (39.09%)	
III	472 (37.73%)	1277 (39.62%)	
IV	263 (21.02%)	686 (21.28%)	
Medical history (%)			
CKD	364 (29.10%)	1021 (31.68%)	0.094
Hypertension	724 (57.87%)	2055 (63.76%)	<0.001
Diabetes	452 (36.13%)	1371 (42.54%)	<0.001
Stroke	262 (20.94%)	881 (27.33%)	<0.001
PAD	124 (9.91%)	421 (13.06%)	0.004
Ischemic etiology	363 (29.02%)	1048 (32.52%)	0.024
CHA_2_DS_2_-VASc score	3.92 ± 1.69	4.30 ± 1.76	<0.001
Laboratory measurements			
WBC (10^9^/L)	6.20 (5.07-7.75)	6.46 (5.16-8.08)	0.007
Hemoglobin (g/L)	132.0 (119.0-145.0)	130.0 (115.2-143.0)	<0.001
Platelets (10^9^/L)	202.0 (161.5-242.5)	198.0 (158.0-244.0)	0.298
ALT (U/L)	23.1 (16.0-36.0)	23.0 (16.0-35.0)	0.579
AST (U/L)	22.0 (17.1-32.3)	22.0 (17.0-31.0)	0.498
Creatinine (µmol/L)	75.9 (63.0-89.2)	76.0 (64.5-91.0)	0.188
eGFR (mL/min/1.73m^2^)	86.0 (67.8-98.3)	83.5 (66.2-96.1)	0.001
FBG (mmol/L)	5.42 (4.69-6.69)	5.60 (4.84-6.96)	<0.001
TG (mmol/L)	1.27 (0.90-1.76)	1.23 (0.87-1.80)	0.225
TC (mmol/L)	3.95 (3.31-4.67)	3.88 (3.22-4.66)	0.185
LDL-C (mmol/L)	2.31 (1.77-2.90)	2.27 (1.73-2.85)	0.304
HDL-C (mmol/L)	1.06 (0.87-1.28)	1.06 (0.88-1.28)	0.763
Potassium (mmol/L)	3.95 (3.68-4.25)	3.96 (3.68-4.28)	0.576
NT-proBNP (pg/mL)	1991.0 (1064.0-3802.5)	1984.0 (1069.0-3972.0)	0.623
IR surrogate indices			
TyG index	8.65 (8.27-9.10)	8.67 (8.26-9.12)	0.540
TyG-BMI index	215.1 (189.4-243.3)	217.7 (193.2-242.5)	0.091
METS-IR	39.0 (33.9-44.5)	39.4 (34.6-44.5)	0.097
TG/HDL-C	1.20 (0.78-1.85)	1.16 (0.75-1.83)	0.287
Echocardiography			
LVEF (%)	50.0 (41.0-57.0)	50.0 (41.0-57.0)	0.726
Medications at discharge (%)			
Antiplatelet agents	518 (41.41%)	1495 (46.39%)	0.003
ACEIs/ARBs/ARNIs	516 (41.25%)	1406 (43.62%)	0.149
Beta-blocker	829 (66.27%)	2214 (68.69%)	0.118
CCB	180 (14.39%)	518 (16.07%)	0.164
Statins	642 (51.32%)	1791 (55.57%)	0.010
DOACs	879 (70.26%)	2368 (73.47%)	0.031
Warfarin	200 (15.99%)	474 (14.71%)	0.283
Digoxin	384 (30.70%)	971 (30.13%)	0.711
MRAs	911 (72.82%)	2299 (71.33%)	0.320
Diuretics	798 (63.79%)	2072 (64.29%)	0.755
SGLT2 inhibitors	115 (9.19%)	342 (10.61%)	0.160
Insulin	82 (6.55%)	249 (7.73%)	0.179
Other oral antidiabetic agents	246 (19.66%)	742 (23.02%)	0.015
Clinical outcome: IS/SE			
Incidence/1000 person-years	16.39	19.17	0.214

*CHF*, chronic heart failure; *AF*, atrial fibrillation; *BMI*, body mass index; *MAP*, mean arterial pressure; *HR*, heart rate; *NYHA*, New York Heart Association; *CKD*, chronic kidney disease; *PAD*, peripheral arterial disease; *PCI*, percutaneous coronary intervention; *CABG* coronary artery bypass grafting; *WBC*, white blood cell; *AST*, aspartate aminotransferase; *ALT* alanine aminotransferase; *eGFR*, estimated glomerular filtration rate; *FBG*, fasting blood glucose; *TG*, triglyceride; *TC*, total cholesterol; *LDL-C*, low-density lipoprotein cholesterol; *HDL-C*, high-density lipoprotein cholesterol; *NT-proBNP*, N-terminal pro-B-type natriuretic peptide; *IR*, insulin resistance; *TyG*, triglyceride-glucose; *TyG-BMI*, triglyceride-glucose body mass index; *METS-IR*, metabolic score for insulin resistance; *TG/HDL-C*, triglyceride to high-density lipoprotein cholesterol ratio; *LVEF*, left ventricular ejection fraction; *ACEI*, angiotensin-converting enzyme inhibitor; *ARB*, angiotensin receptor blocker; *ARNI*, angiotensin receptor-neprilysin inhibitor; *CCB*, calcium channel blocker; *DOACs*, direct oral anticoagulants; *MRAs*, mineralocorticoid receptor antagonists; *SGLT2, inhibitors* sodium-glucose cotransporter 2 inhibitors; *IS/SE*, ischemic stroke/systemic embolism. Ischemic etiology was defined as a history of myocardial infarction, prior PCI, or prior CABG.

Patients with non-paroxysmal AF had higher white blood cell count and FBG levels but lower hemoglobin levels and estimated glomerular filtration rate than those with paroxysmal AF (all *P* < 0.05). However, lipid profiles, N-terminal pro-B-type natriuretic peptide, left ventricular ejection fraction, and the four IR surrogate indices were generally similar between the two groups. At discharge, antiplatelet agents, statins, direct oral anticoagulants, and other oral antidiabetic agents were more frequently used in patients with non-paroxysmal AF. Additional comparisons of baseline characteristics according to tertiles of the TyG index, TyG-BMI index, METS-IR, and TG/HDL-C ratio are presented in **Supplementary Tables S2–S5**, respectively.

The incidence rate of IS/SE was numerically higher in the non-paroxysmal AF group, but the difference was not statistically significant (19.17 vs. 16.39 per 1,000 person-years, *P* = 0.214).

### Associations of IR surrogate indices with IS/SE

The incidence of IS/SE increased progressively across tertiles of all IR surrogate indices (**Table 2**). Kaplan–Meier analysis demonstrated significant differences in IS/SE-free survival across tertiles of the TyG index, TyG-BMI index, METS-IR, and TG/HDL-C ratio (**Figure 3**, log-rank test, all *P* < 0.001). RCS analyses further showed positive associations between higher levels of the IR surrogate indices and increased IS/SE risk, with significant overall associations observed for all four indices (**Figure 4**, all *P* for overall association < 0.001).

**Table 2:** Associations between IR surrogate indices and IS/SE in patients with CHF and AF.

Categories	Incidence/ 1000 person-y	Unadjusted			Model 1			Model 2		
		HR (95% CI)	*P*-value	*P* for trend	HR (95% CI)	*P*-value	*P* for trend	HR (95% CI)	*P*-value	*P* for trend
TyG index										
Per 1-SD increase		1.35 (1.22-1.49)	**<0.001**		1.36 (1.23-1.50)	**<0.001**		1.27 (1.13-1.43)	**<0.001**	
Tertiles^a^				**<0.001**			**<0.001**			**0.004**
T1 (n=1491)	12.73	Ref.			Ref.			Ref.		
T2 (n=1491)	18.59	1.46 (1.11-1.93)	**0.007**		1.46 (1.11-1.93)	**0.008**		1.34 (1.00-1.78)	**0.049**	
T3 (n=1492)	24.47	1.94 (1.49-2.55)	**<0.001**		1.96 (1.50-2.57)	**<0.001**		1.58 (1.17-2.14)	**0.003**	
TyG-BMI index										
Per 1-SD increase		1.46 (1.33-1.59)	**<0.001**		1.45 (1.33-1.58)	**<0.001**		1.41 (1.28-1.56)	**<0.001**	
Tertiles^a^				**<0.001**			**<0.001**			**<0.001**
T1 (n=1491)	9.82	Ref.			Ref.			Ref.		
T2 (n=1491)	15.92	1.62 (1.19-2.22)	**0.002**		1.62 (1.19-2.22)	**0.003**		1.66 (1.21-2.29)	**0.002**	
T3 (n=1492)	29.98	3.07 (2.30-4.09)	**<0.001**		3.08 (2.31-4.10)	**<0.001**		2.97 (2.20-4.02)	**<0.001**	
METS-IR										
Per 1-SD increase		1.24 (1.18-1.30)	**<0.001**		1.23 (1.18-1.29)	**<0.001**		1.27 (1.20-1.34)	**<0.001**	
Tertiles^a^				**<0.001**			**<0.001**			**<0.001**
T1 (n=1491)	9.10	Ref.			Ref.			Ref.		
T2 (n=1491)	15.78	1.74 (1.26-2.40)	**<0.001**		1.76 (1.28-2.43)	**<0.001**		1.79 (1.29-2.48)	**<0.001**	
T3 (n=1492)	30.79	3.39 (2.53-4.54)	**<0.001**		3.53 (2.64-4.73)	**<0.001**		3.64 (2.69-4.92)	**<0.001**	
TG/HDL-C										
Per 1-SD increase		1.11 (1.06-1.16)	**<0.001**		1.11 (1.06-1.15)	**<0.001**		1.13 (1.08-1.19)	**<0.001**	
Tertiles^a^				**<0.001**			**<0.001**			**<0.001**
T1 (n=1491)	12.38	Ref.			Ref.			Ref.		
T2 (n=1491)	18.65	1.50 (1.13-1.99)	**0.005**		1.54 (1.16-2.04)	**0.003**		1.56 (1.17-2.09)	**0.002**	
T3 (n=1492)	24.29	1.96 (1.49-2.58)	**<0.001**		2.07 (1.58-2.73)	**<0.001**		1.96 (1.48-2.60)	**<0.001**	

*IR*, insulin resistance; *IS/SE*, ischemic stroke/systemic embolism; *CHF*, chronic heart failure; *AF*, atrial fibrillation; *TyG*, triglyceride-glucose; *TyG-BMI*, triglyceride-glucose body mass index; *METS-IR*, metabolic score for insulin resistance; *TG/HDL-C*, triglyceride to high-density lipoprotein cholesterol ratio; *HR*, hazard ratio; *CI*, confidence interval; *SD*, standard deviation..

Model 1: adjusted for age, sex, and smoking status.

Model 2: adjusted for Model 1 plus MAP, heart rate, NYHA class, LVEF, NT-proBNP, LDL-C, WBC count, ALT, serum creatinine, diabetes mellitus, hypertension, prior stroke, PAD, ischemic etiology, anticoagulants, antiplatelet agents, statins, ACEIs/ARBs/ARNIs, beta-blockers, MRAs, digoxin, SGLT2 inhibitors, other glucose-lowering medications, and, where applicable, BMI, FBG, TG, and HDL-C (excluding components of the corresponding IR index).

^a^ Tertiles were defined according to the distribution of each IR surrogate index. *P* values < 0.05 are presented in bold.

**Figure 3:**
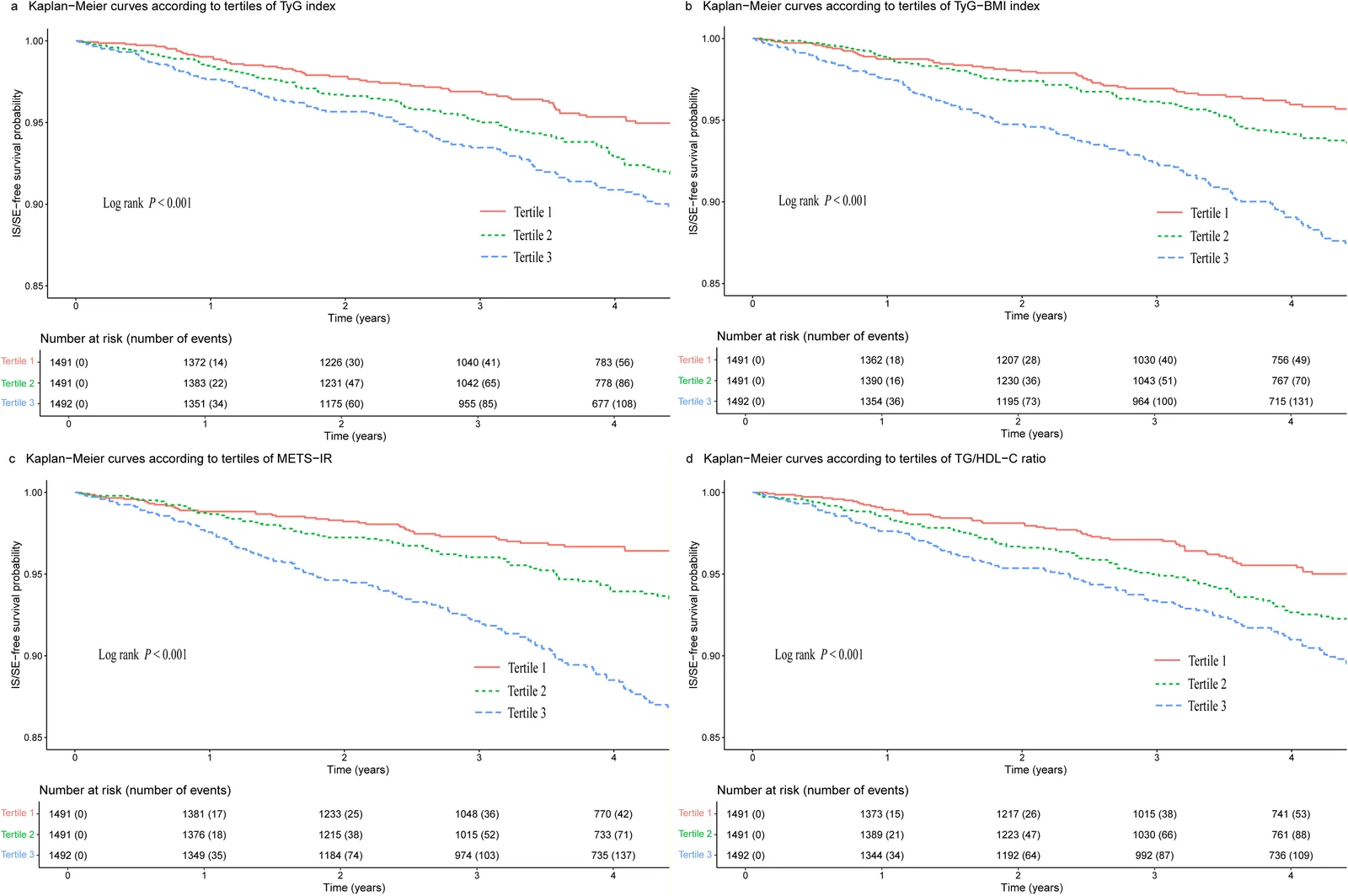
Kaplan–Meier curves for IS/SE-free survival across tertiles of IR surrogate indices in patients with CHF and AF. *IR*, insulin resistance; *IS/SE*, ischemic stroke/systemic embolism; *CHF*, chronic heart failure; *AF*, atrial fibrillation; TyG index, triglyceride-glucose index. *TyG-BMI*, triglyceride-glucose body mass index; *METS-IR*, metabolic score for insulin resistance; *TG/HDL-C*, triglyceride to high-density lipoprotein cholesterol ratio.

**Figure 4:**
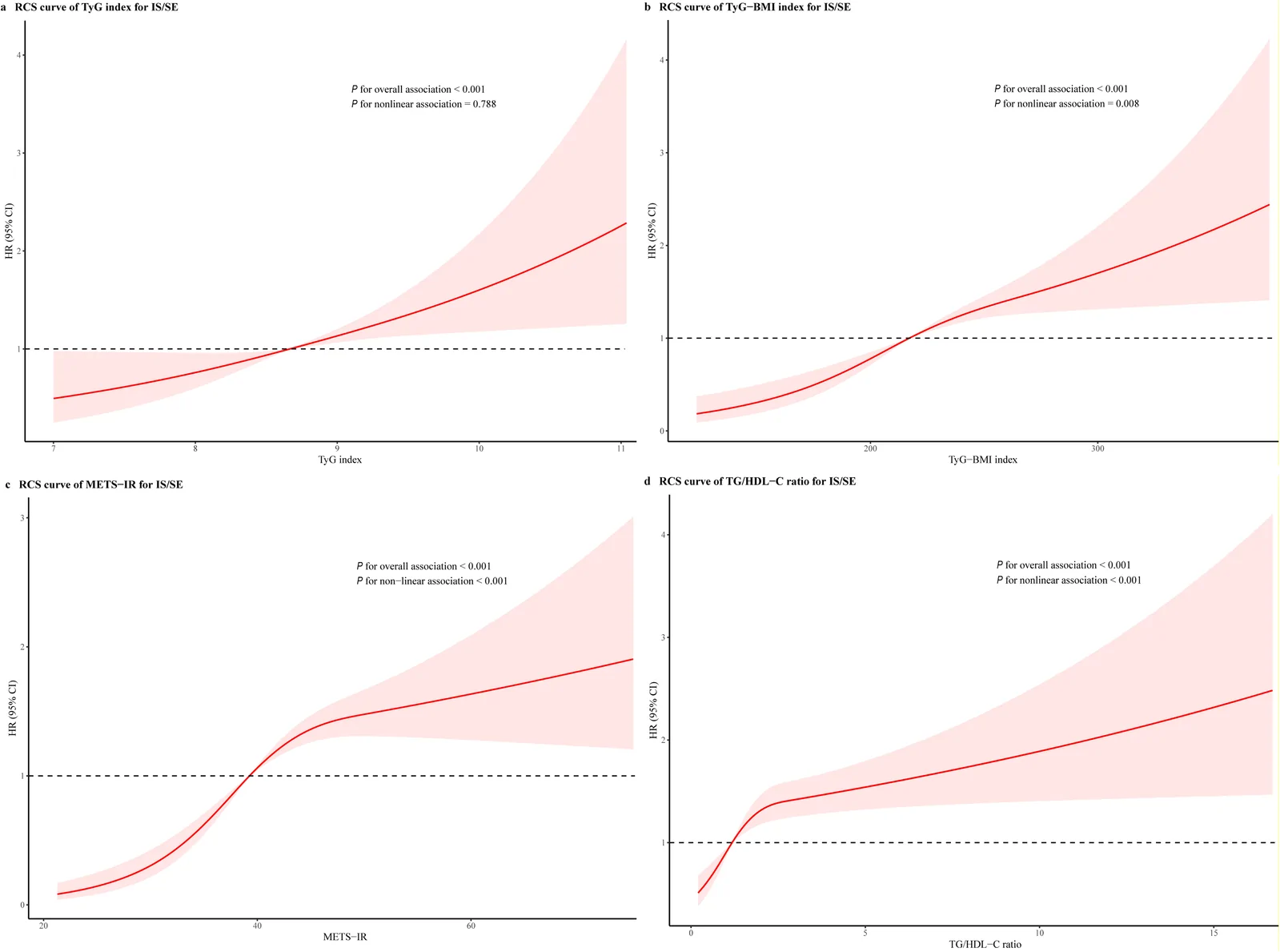
HRs for IS/SE by IR surrogate indices from RCS analyses adjusted for Model 2. *RCS*, restricted cubic spline; *HR*, hazard ratio; *CI*, confidence interval; *IS/SE*, ischemic stroke/systemic embolism; *IR*, insulin resistance; TyG index, triglyceride-glucose index. *TyG-BMI*, triglyceride-glucose body mass index; *METS-IR*, metabolic score for insulin resistance; *TG/HDL-C*, triglyceride to high-density lipoprotein cholesterol ratio.

In multivariable Cox regression analyses, all four IR surrogate indices were significantly associated with IS/SE. After full adjustment in Model 2, each 1-SD increase in the TyG index, TyG-BMI index, METS-IR, and TG/HDL-C ratio was associated with a higher risk of IS/SE, with HRs of 1.27 (95% CI, 1.13–1.43), 1.41 (1.28–1.56), 1.27 (1.20–1.34), and 1.13 (1.08–1.19), respectively (all *P* < 0.001). Consistent results were observed when these indices were analyzed as tertiles, with the highest tertiles showing significantly higher IS/SE risk than the lowest tertiles (*P* for trend = 0.004 for the TyG index and <0.001 for the other indices) (**Table 2**). Diabetes-stratified analyses showed similar findings, with the highest tertile of each index associated with increased IS/SE risk in both subgroups (**Supplementary Figure S1**). Fine–Gray analyses treating death before IS/SE as a competing event yielded results consistent with the primary analyses (**Supplementary Table S6**).

### Associations of IR surrogate indices with IS/SE stratified by AF type

After stratification by AF type, the associations were generally directionally consistent, although statistical significance was less consistent in patients with paroxysmal AF (**Supplementary Table S7** and **Figure 5**). In the fully adjusted model, the highest tertiles of the TyG-BMI index, METS-IR, and TG/HDL-C ratio were significantly associated with increased IS/SE risk in patients with paroxysmal AF (HRs, 2.50–3.18), whereas the association for the TyG index did not reach statistical significance (HR, 1.79; 95% CI, 0.95–3.40; *P* = 0.073). In patients with non-paroxysmal AF, the highest tertiles of all four indices were significantly associated with increased IS/SE risk (HRs, 1.63–3.72). Significant trends were observed for the TyG-BMI index, METS-IR, and TG/HDL-C ratio in paroxysmal AF and for all four indices in non-paroxysmal AF. Kaplan–Meier and RCS analyses generally supported these findings, although the overall RCS association for the TyG index was not significant in paroxysmal AF (*P* = 0.383) (**Supplementary Figures S2**, **S3**).

**Figure 5:**
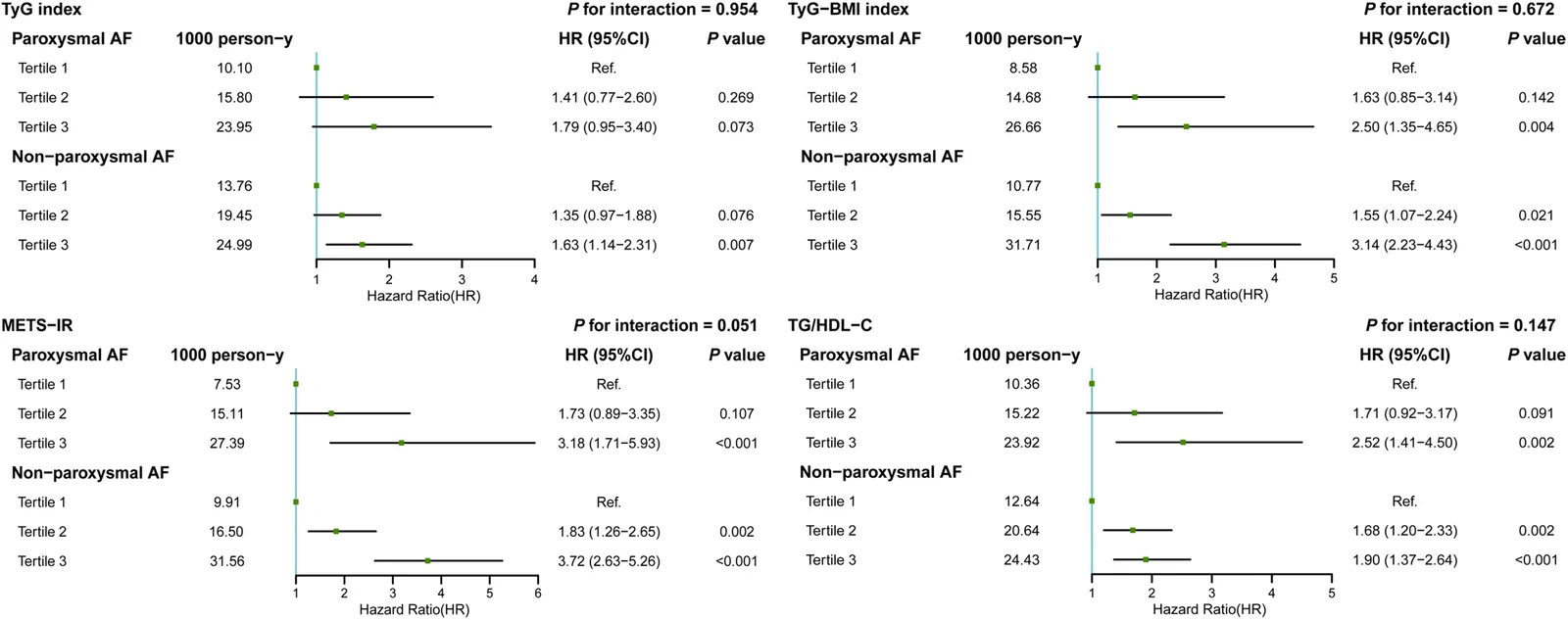
Forest plots of IS/SE risk across subgroup-specific tertiles of IR surrogate indices, stratified by AF type and adjusted for covariates in Model 2. *IR*, insulin resistance; *IS/SE*, ischemic stroke/systemic embolism; *AF*, atrial fibrillation; *TyG index*, triglyceride-glucose index; *TyG-BMI*, triglyceride-glucose body mass index; *METS-IR*, metabolic score for insulin resistance; *TG/HDL-C*, triglyceride to high-density lipoprotein cholesterol ratio; *HR*, hazard ratio; *CI*, confidence interval. A *P* value for interaction <0.10 was considered suggestive of potential heterogeneity.

Notably, the risk gradients were more consistently observed in patients with non-paroxysmal AF, in whom significant associations from the second tertile onward were observed for the TyG-BMI index, METS-IR, and TG/HDL-C ratio, whereas the TyG index was significantly associated with IS/SE only in the highest tertile. In patients with paroxysmal AF, significant associations were confined to the highest tertiles of the TyG-BMI index, METS-IR, and TG/HDL-C ratio (**Supplementary Table S7** and **Figure 5**). Interaction analysis suggested potential heterogeneity only for METS-IR (*P* for interaction = 0.051), with no evidence of interaction for the TyG index, TyG-BMI index, or TG/HDL-C ratio (*P* for interaction = 0.954, 0.672, and 0.147, respectively). Thus, although the risk gradients appeared more consistent in patients with non-paroxysmal AF, formal evidence of effect modification by AF type was limited, and these findings should be considered exploratory.

### Incremental predictive value beyond the CHA_2_DS_2_-VASc score

The incremental predictive value of IR surrogate indices beyond the CHA_2_DS_2_-VASc score for 4-year IS/SE risk is shown in **Table 3** and **Figure 6**. The CHA_2_DS_2_-VASc score alone yielded an AUC of 0.637 (95% CI, 0.601–0.672). Compared with the CHA_2_DS_2_-VASc score alone, adding the TyG-BMI index, METS-IR, or TG/HDL-C ratio significantly improved the AUC to 0.673, 0.682, and 0.655, respectively, whereas the improvement after adding the TyG index was not statistically significant (AUC, 0.651; *P* = 0.276). In reclassification analyses, the TyG index, TyG-BMI index, and METS-IR significantly improved both NRI and IDI, whereas the TG/HDL-C ratio mainly improved IDI, with only a borderline improvement in NRI (*P* = 0.060). Among the models incorporating different IR surrogate indices, METS-IR achieved the highest AUC and showed significantly better performance than the TyG index and TG/HDL-C ratio in pairwise AUC comparisons, although its advantage over the TyG-BMI index was not statistically significant (*P* = 0.326). DCA further indicated that models incorporating IR surrogate indices, particularly the TyG-BMI index and METS-IR, provided greater net benefit than the CHA_2_DS_2_-VASc score alone.

**Table 3:** Incremental predictive value of IR surrogate indices beyond the CHA_2_DS_2_-VASc score for IS/SE in patients with CHF and AF.

Model	AUC (95% CI)	*P* value	NRI (95% CI)	*P* value	IDI (95% CI)	*P* value
CHA_2_DS_2_-VASc score	0.637 (0.601-0.672)	Ref.	–	Ref.	–	Ref.
+ TyG index	0.651 (0.616-0.687)	0.276	0.205 (0.100-0.347)	<0.001	0.006 (0.002-0.012)	<0.001
+ TyG-BMI index	0.673 (0.640-0.706)	0.018	0.386 (0.232-0.513)	<0.001	0.010 (0.005-0.018)	<0.001
+ METS-IR	0.682 (0.649-0.715)	<0.001	0.408 (0.215-0.519)	0.016	0.005 (0.001-0.008)	0.016
+ TG/HDL-C	0.655 (0.619-0.690)	0.001	0.201 (-0.025-0.315)	0.060	0.002 (0.000-0.004)	0.048
Pairwise comparison of AUC						
+ TyG index vs + TyG-BMI index	0.651 vs 0.673	0.133	-	-	-	-
+ TyG index vs + METS-IR	0.651 vs 0.682	0.021	-	-	-	-
+ TyG index vs + TG/HDL-C	0.651 vs 0.655	0.752	-	-	-	-
+ TyG-BMI index vs + METS-IR	0.673 vs 0.682	0.326	-	-	-	-
+ TyG-BMI index vs + TG/HDL-C	0.673 vs 0.655	0.237	-	-	-	-
+ METS-IR vs + TG/HDL-C	0.682 vs 0.655	0.003	-	-	-	-

*IR*, insulin resistance; *IS/SE*, ischemic stroke/systemic embolism; *CHF*, chronic heart failure; *AF*, atrial fibrillation; *TyG*, triglyceride-glucose; *TyG-BMI*, triglyceride-glucose body mass index; *METS-IR*, metabolic score for insulin resistance; *TG/HDL-C*, triglyceride to high-density lipoprotein cholesterol ratio; *AUC*, area under the receiver operating characteristic curve; *CI*, confidence interval; *NRI*, net reclassification improvement; *IDI*, integrated discrimination improvement. Predictive performance was evaluated for 4-year IS/SE risk. “+” indicates that the corresponding IR surrogate index was added to the CHA_2_DS_2_-VASc score. *P* values for AUC, NRI, and IDI indicate comparisons with the CHA_2_DS_2_-VASc score model. Pairwise comparisons of AUC were performed among models incorporating different IR surrogate indices..

**Figure 6:**
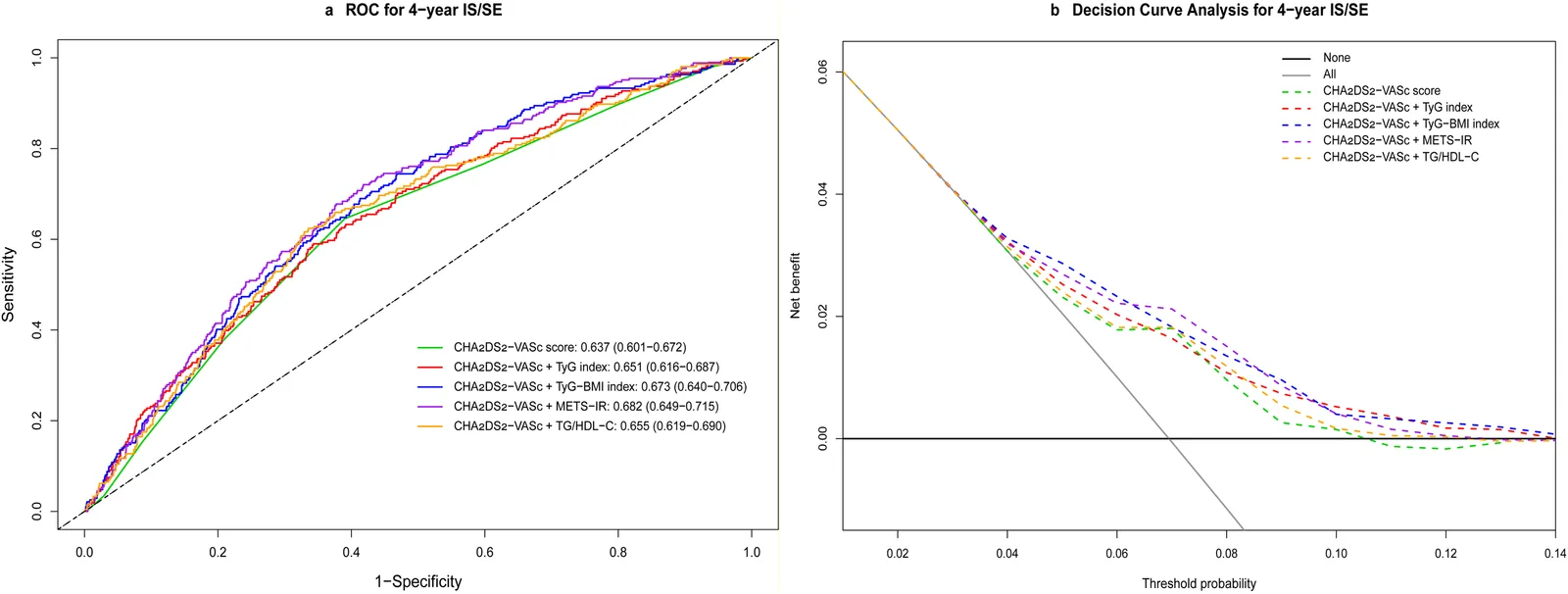
Incremental predictive performance of IR surrogate indices beyond the CHA_2_DS_2_-VASc score for IS/SE prediction in patients with CHF and AF. *ROC*, receiver operating characteristic; *DCA*, decision curve analysis; *IR*, insulin resistance; *IS/SE*, ischemic stroke/systemic embolism; *CHF*, chronic heart failure; *AF*, atrial fibrillation; *TyG, index* triglyceride-glucose index; *TyG-BMI*, triglyceride-glucose body mass index; *METS-IR*, metabolic score for insulin resistance; *TG/HDL-C*, triglyceride to high-density lipoprotein cholesterol ratio.

### Incremental predictive value beyond the CHA_2_DS_2_-VASc score according to AF type

The incremental predictive value of IR surrogate indices was further examined separately according to AF type (**Supplementary Table S8** and **Supplementary Figures S4**, **S5**). In patients with paroxysmal AF, adding IR surrogate indices numerically increased the AUC from 0.687 to 0.692–0.716, but none of the AUC improvements reached statistical significance (all *P* > 0.05). Nevertheless, all four indices significantly improved NRI (all *P* < 0.05), and all except the TyG index significantly improved IDI.

In patients with non-paroxysmal AF, adding the TyG-BMI index, METS-IR, or TG/HDL-C ratio significantly improved the AUC from 0.617 to 0.661, 0.672, and 0.635, respectively (all *P* < 0.05), whereas adding the TyG index did not (AUC, 0.637; *P* = 0.263). Reclassification improvement was more consistent for the TyG index, TyG-BMI index, and METS-IR, whereas the TG/HDL-C ratio showed only borderline improvements in NRI and IDI. METS-IR achieved the highest AUC and outperformed the TyG index and TG/HDL-C ratio, but not the TyG-BMI index, in pairwise AUC comparisons. DCA further indicated greater net benefit after adding IR surrogate indices, particularly the TyG-BMI index and METS-IR, with more evident improvement in non-paroxysmal AF.

## Discussion

In this study, we evaluated the associations of IR surrogate indices with the risk of IS/SE in patients with CHF and AF, and further assessed their incremental predictive value beyond the CHA_2_DS_2_-VASc score. The principal findings were as follows. First, higher levels of IR surrogate indices were associated with an increased risk of IS/SE. Second, the addition of IR surrogate indices provided incremental predictive value beyond the CHA_2_DS_2_-VASc score, suggesting that IR-related metabolic burden may capture risk information not fully reflected by conventional clinical variables. Third, exploratory AF-type-stratified analyses suggested that the incremental predictive improvements were more evident in patients with non-paroxysmal AF. These findings suggest that readily available IR surrogate indices may complement CHA_2_DS_2_-VASc-based thromboembolic risk assessment in patients with CHF and AF, particularly among those with non-paroxysmal AF.

Thromboembolic events are among the most serious complications of AF, and their prevention remains a central component of AF management (15). This issue is particularly relevant in patients with concomitant CHF and AF, because these two conditions frequently coexist and may aggravate each other through shared pathophysiological pathways, including atrial pressure and volume overload, neurohormonal activation, inflammation, endothelial dysfunction, and atrial remodeling (1, 19). In this setting, AF-related atrial mechanical dysfunction and blood stasis may interact with the hemodynamic and systemic abnormalities of CHF, thereby contributing to a vulnerable thromboembolic substrate. Once ischemic stroke occurs, it may cause substantial neurological injury, worsen functional status, and increase adverse outcomes; in patients with concomitant HF, such events may further complicate long-term disease management (2, 20). Therefore, more refined identification of patients at increased thromboembolic risk is clinically important in the CHF-AF population.

The CHA_2_DS_2_-VASc score remains a well-established and widely used clinical tool for thromboembolic risk assessment in patients with AF (3, 16). However, this score is primarily based on conventional demographic and clinical risk factors, including age, HF, hypertension, diabetes mellitus, prior stroke or thromboembolism, vascular disease, and sex (16). It does not directly incorporate metabolic abnormalities, IR, lipid-related risk, inflammatory status, or obesity-related metabolic burden, all of which may contribute to thromboembolic risk in patients with CHF and AF. Consistently, a previous systematic review and meta-analysis showed that the discriminatory performance of the CHA_2_DS_2_-VASc score for predicting ischemic stroke was modest, suggesting that additional markers may be needed to improve risk stratification (4). The development of other risk tools, such as the biomarker-based ABC-stroke score and the GARFIELD-AF risk tool, further reflects the ongoing effort to refine stroke and systemic embolism risk prediction beyond conventional clinical scores (21, 22). Therefore, exploring readily available indicators that can complement CHA_2_DS_2_-VASc-based assessment may be particularly meaningful in the CHF-AF population.

IR may be a clinically relevant metabolic factor in this context. As a central feature of metabolic dysfunction, IR is closely related to dyslipidemia, low-grade inflammation, endothelial dysfunction, obesity, and hypertension, all of which may contribute to cardiovascular risk (23). In patients with HF, abnormalities in cardiac energy metabolism and systemic metabolic regulation are increasingly recognized as important contributors to disease progression (24). In patients with AF, IR has been associated with arrhythmogenesis, atrial structural and electrical remodeling, and a higher likelihood of AF occurrence or recurrence (7, 25). These links suggest that IR may represent a shared metabolic substrate connecting CHF, AF, and thromboembolic risk. Because direct assessment of IR is not routinely feasible in clinical practice, surrogate indices derived from routine laboratory and anthropometric parameters, including the TyG index, TyG-BMI index, METS-IR, and TG/HDL-C ratio, have attracted attention as simple and accessible markers of IR-related metabolic burden (8–11). The consistent associations observed across these indices in our study further support the relevance of IR-related metabolic abnormalities in patients with CHF and AF.

The association between IR surrogate indices and IS/SE risk may be explained by several mechanisms. IR can impair insulin-mediated endothelial nitric oxide signaling and promote oxidative stress, vascular inflammation, atherosclerosis, and endothelial dysfunction, thereby increasing susceptibility to ischemic events (5, 23). IR-related metabolic disturbances may also contribute to a prothrombotic state through increased platelet reactivity, enhanced coagulation activation, and impaired fibrinolysis (5, 26). In addition, IR-related dyslipidemia and adiposity may accelerate atherosclerotic progression and further increase vascular vulnerability (5, 6, 23). In patients with CHF and AF, these metabolic and vascular abnormalities may interact with AF-related atrial mechanical dysfunction, blood stasis, and HF-related neurohormonal and inflammatory activation, thereby amplifying thromboembolic vulnerability (1, 7, 19). The graded associations across tertiles and the dose–response patterns observed in our analyses are consistent with the concept that increasing IR-related metabolic burden is progressively linked to higher IS/SE risk.

The incremental predictive value observed in our study further supports the potential role of IR surrogate indices as complementary tools for thromboembolic risk assessment. In the overall cohort, the CHA_2_DS_2_-VASc score showed only moderate discrimination for IS/SE risk, with an AUC of 0.637, which is consistent with previous evidence showing the modest predictive performance of conventional clinical risk scores in AF (4, 27). The addition of several IR surrogate indices, particularly the TyG-BMI index and METS-IR, improved model performance, as shown by higher discrimination, improved reclassification, and greater clinical net benefit. These findings suggest that IR-related metabolic burden may capture residual risk not fully represented by conventional clinical variables. Notably, indices incorporating both glycemic–lipid information and obesity-related burden, such as the TyG-BMI index and METS-IR, appeared to provide more evident incremental information. This finding is biologically plausible because these composite indices may better reflect multidimensional metabolic dysfunction, adiposity-related burden, and IR-related cardiometabolic risk (8–10). Together with recent evidence showing that the TyG index can predict thrombotic events in patients with AF, our findings suggest that easily obtainable metabolic indices may help complement, rather than replace, CHA_2_DS_2_-VASc-based assessment in patients with CHF and AF (28).

The AF-type-stratified findings should be interpreted as clinically motivated and exploratory. Associations were generally consistent across AF types, although the TyG index was not significant in paroxysmal AF after full adjustment. Risk gradients and incremental improvements were more evident in non-paroxysmal AF, particularly for the TyG-BMI index and METS-IR. This pattern is biologically plausible because non-paroxysmal AF may reflect a more sustained arrhythmic phenotype and a more advanced atrial cardiomyopathic substrate characterized by structural remodeling, impaired atrial mechanical function, and blood stasis (12, 13, 29, 30). In this setting, IR-related inflammation, oxidative stress, fibrotic signaling, endothelial dysfunction, and prothrombotic activation may further amplify thromboembolic vulnerability (5, 7, 23, 26). However, formal interaction analysis suggested potential heterogeneity only for METS-IR (*P* for interaction = 0.051), whereas no evidence of interaction was observed for the TyG index, TyG-BMI index, or TG/HDL-C ratio (*P* for interaction = 0.954, 0.672, and 0.147, respectively). Moreover, differences in subgroup size and event distribution, together with the lower discrimination of the baseline CHA_2_DS_2_-VASc model in patients with non-paroxysmal AF, may also have contributed to the more apparent incremental improvement observed in this subgroup. Clinical AF classification is also an imperfect surrogate for quantitative AF burden. Therefore, these findings do not establish definitive subtype-specific effects but should instead be considered hypothesis-generating and require validation in prospective studies incorporating continuous rhythm monitoring and more detailed atrial phenotyping.

### Strengths and limitations

This study has several strengths. First, it focused on patients with concomitant CHF and AF, a clinically important population with a high thromboembolic risk, and was based on a relatively large real-world cohort. Second, four commonly used IR surrogate indices were comprehensively evaluated, providing a broader assessment of IR-related metabolic burden. Third, the study not only examined the associations between IR surrogate indices and IS/SE using multiple analytical approaches but also assessed their incremental predictive value beyond the CHA_2_DS_2_-VASc score. Finally, clinically motivated exploratory analyses according to AF type generated hypotheses regarding potential heterogeneity in the prognostic relevance of IR-related metabolic burden across different AF phenotypes.

Several limitations should be acknowledged. First, this was a retrospective observational study; therefore, residual confounding cannot be excluded, and causal relationships cannot be established. Second, IR was assessed using surrogate indices derived from routine clinical variables, whereas fasting insulin and reference-standard measures, such as the hyperinsulinemic–euglycemic clamp, were unavailable. Third, only baseline clinical data and discharge medications were available, precluding assessment of longitudinal changes and confirmation of sustained guideline-concordant anticoagulation. Fourth, AF type was determined from clinical records without continuous rhythm monitoring, precluding quantitative assessment of AF burden. The AF-type-stratified analyses were also exploratory and involved unequal subgroup sizes and multiple comparisons; therefore, these findings should be considered hypothesis-generating. Fifth, the limited sample size and event count in the intermediate-risk subgroup precluded reliable interaction and risk reclassification analyses. Sixth, because most patients were prescribed oral anticoagulation at discharge, the observed IS/SE events likely reflect residual thromboembolic risk in a cohort with high baseline anticoagulant use rather than untreated baseline risk. Therefore, these findings should not be directly extrapolated to decisions regarding the initiation of anticoagulation.

## Conclusions

In patients with CHF and AF, IR surrogate indices were associated with increased IS/SE risk, while the TyG-BMI index and METS-IR provided the most consistent incremental predictive value beyond the CHA_2_DS_2_-VASc score. These improvements appeared more evident in non-paroxysmal AF, although the subgroup findings were exploratory and require prospective validation.

## Data Availability

The raw data supporting the conclusions of this article will be made available by the authors, without undue reservation.

## Supplementary material

Supplementary Figure 1 Forest plots of IS/SE risk across tertiles of IR surrogate indices stratified by diabetes status adjusted for Model 2.

Supplementary Figure 2 Kaplan–Meier curves for IS/SE-free survival by tertiles of IR surrogate indices stratified by AF type.

Supplementary Figure 3 HRs for IS/SE by IR surrogate indices across AF-type subgroups from restricted cubic spline analyses adjusted for Model 2.

Supplementary Figure 4 ROC curves evaluating the incremental effect of IR surrogate indices beyond the CHA_2_DS_2_-VASc score for IS/SE stratified by AF type.

Supplementary Figure 5 Decision curve analysis comparing the CHA_2_DS_2_-VASc score with models incorporating IR surrogate indices for IS/SE prediction stratified by AF type.

Supplementary Table 1 Univariable Cox regression analysis of factors associated with IS/SE.

Supplementary Table 2 **-S5.** Baseline characteristics across tertiles of the four IR indices.

Supplementary Table 3 Baseline characteristics across tertiles of the four IR indices.

Supplementary Table 4 Baseline characteristics across tertiles of the four IR indices.

Supplementary Table 5 Baseline characteristics across tertiles of the four IR indices.

Supplementary Table 6 Associations between IR surrogate indices and IS/SE accounting for all-cause death as a competing risk.

Supplementary Table 7 Associations between IR surrogate indices and IS/SE stratified by AF type.

Supplementary Table 8 Incremental predictive value of IR surrogate indices beyond the CHA_2_DS_2_-VASc score for IS/SE stratified by AF type.
