# Supplementary material for: Incremental value of insulin resistance surrogate indices beyond the CHA2DS2-VASc score for predicting thromboembolic events in patients with chronic heart failure and atrial fibrillation
Source: Front Endocrinol (Lausanne). 2026 Sep 23;17:1954348. doi: 10.3389/fendo.2026.1954348 (PMC13641996; doi:10.3389/fendo.2026.1954348)
Supplement: Supplementary file 1 [file DataSheet1.pdf]

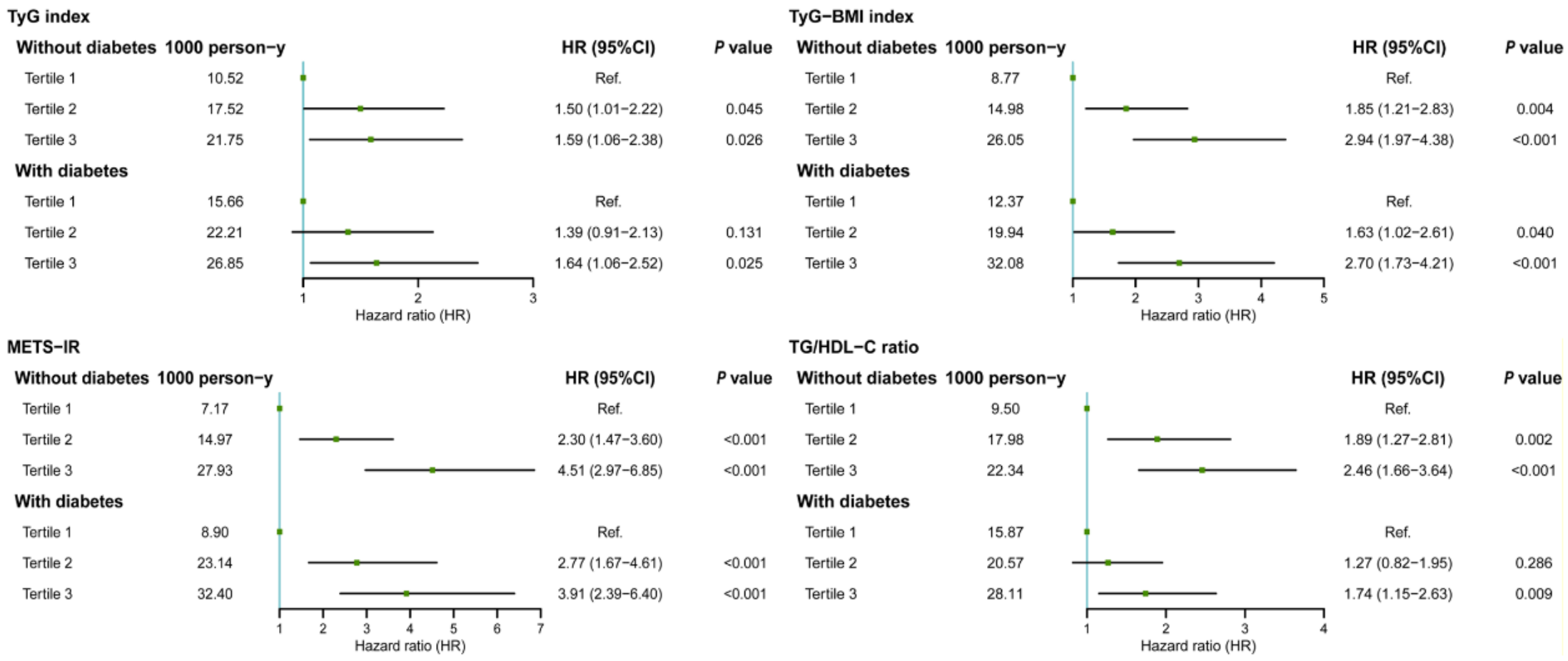

Fig. S1 Forest plots of IS/SE risk across tertiles of IR surrogate indices, stratified by diabetes status and adjusted for Model 2. Within each diabetes-status subgroup, tertiles were defined according to the subgroup-specific distribution of each IR surrogate index, with T1 as the reference. *IR* insulin resistance, *IS/SE* ischemic stroke/systemic embolism, *TyG index* triglyceride-glucose index, *TyG-BMI* triglyceride-glucose body mass index, *METS-IR* metabolic score for insulin resistance, *TG/HDL-C* triglyceride to high-density lipoprotein cholesterol ratio, *HR* hazard ratio, *CI* confidence interval.

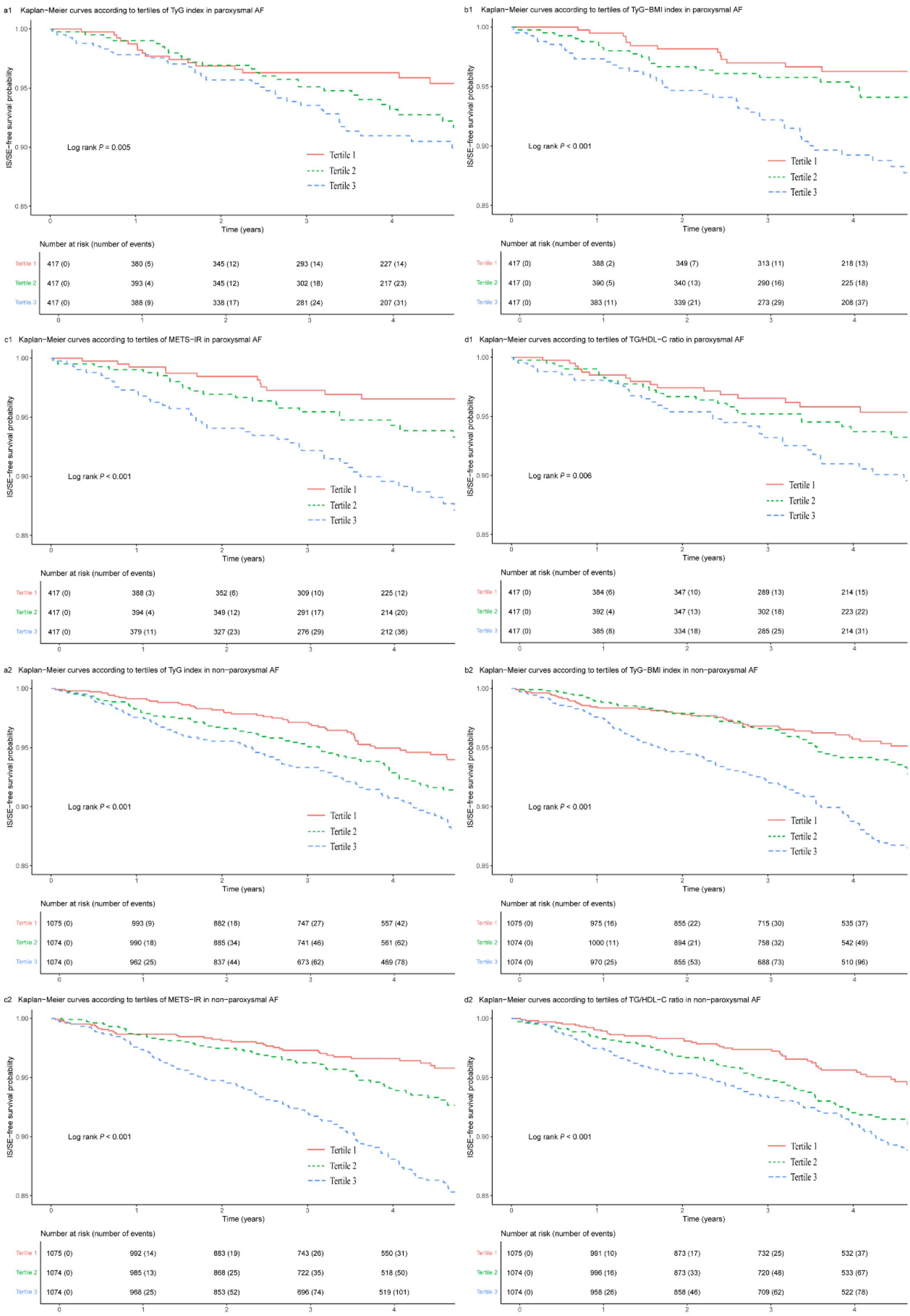

Fig. S2 Kaplan–Meier curves for IS/SE-free survival across subgroup-specific tertiles of IR surrogate indices, stratified by AF type. Within each AF-type subgroup, tertiles were defined according to the subgroup-specific distribution of each IR surrogate index. *TyG index* triglyceride-glucose index, *TyG-BMI* triglyceride-glucose body mass index, *METS-IR* metabolic score for insulin resistance, *TG/HDL-C* triglyceride to high-density lipoprotein cholesterol ratio.

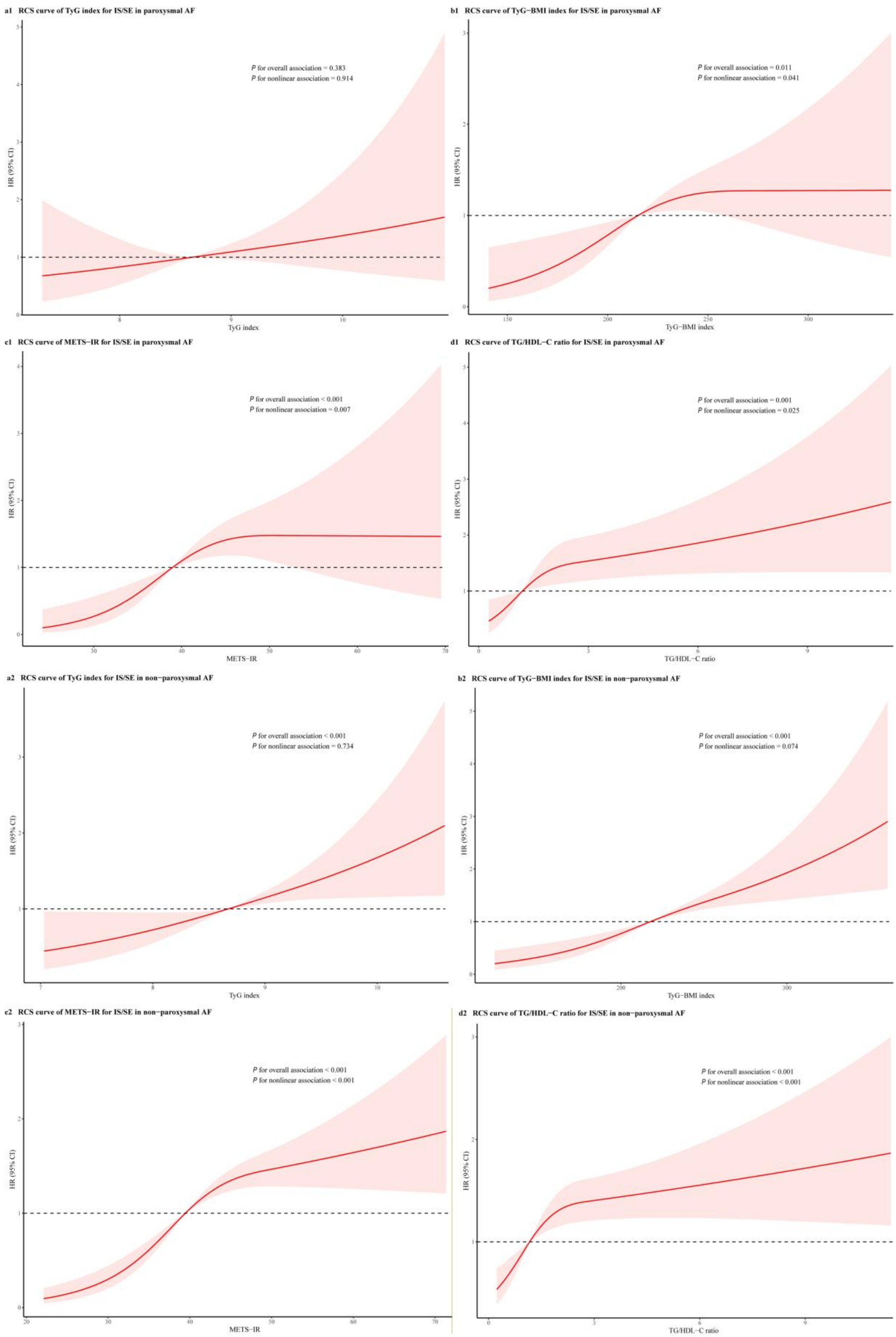

Fig. S3 HRs for IS/SE by IR surrogate indices across AF-type subgroups from restricted cubic spline analyses adjusted for Model 2. *IR* insulin resistance, *IS/SE* ischemic stroke/systemic embolism, *AF* atrial fibrillation, *TyG index* triglyceride-glucose index, *TyG-BMI* triglyceride-glucose body mass index, *METS-IR* metabolic score for insulin resistance, *TG/HDL-C* triglyceride to high-density lipoprotein cholesterol ratio, *HR* hazard

ratio.

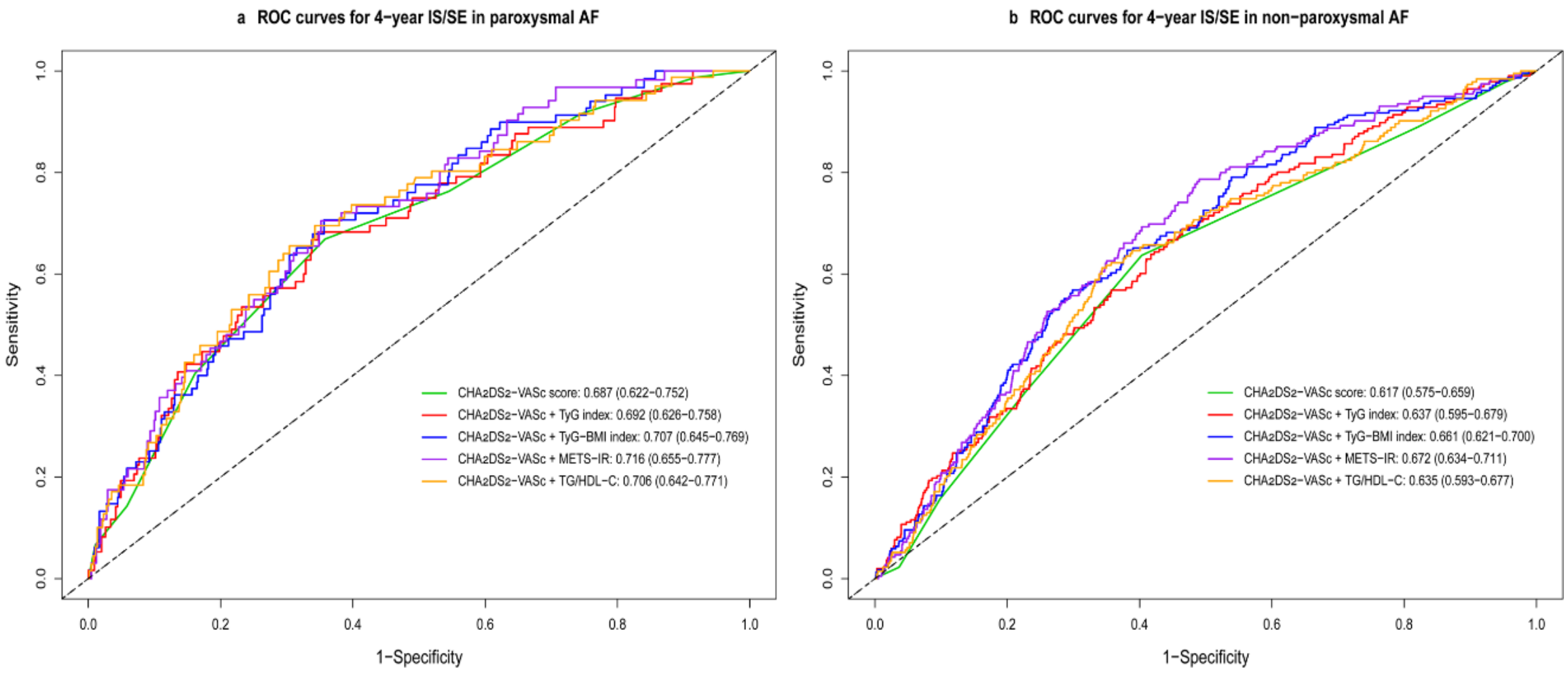

Fig. S4 ROC curves evaluating the incremental effect of IR surrogate indices beyond the CHA_2DS_2-VASc score for IS/SE stratified by AF type. *ROC* receiver operating characteristic, *IR* insulin resistance, *IS/SE* ischemic stroke/systemic embolism, *AF* atrial fibrillation, *TyG index* triglyceride-glucose index, *TyG-BMI* triglyceride-glucose body mass index, *METS-IR* metabolic score for insulin resistance, *TG/HDL-C* triglyceride to high-density lipoprotein cholesterol ratio.

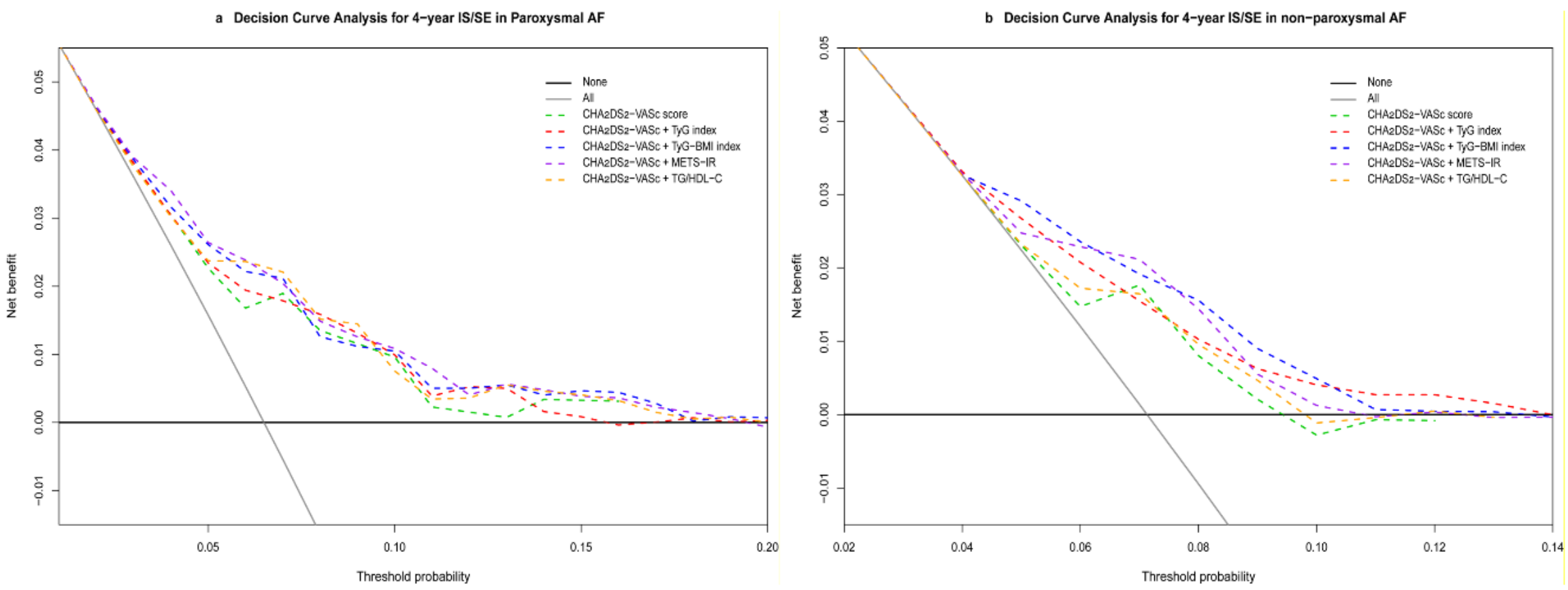

Fig. S5 Decision curve analysis comparing the CHA_2DS_2-VASc score with models incorporating IR surrogate indices for IS/SE prediction stratified by AF type. *IR* insulin resistance, *IS/SE* ischemic stroke/systemic embolism, *AF* atrial fibrillation, *TyG index* triglyceride-glucose index, *TyG-BMI* triglyceride-glucose body mass index, *METS-IR* metabolic score for insulin resistance, *TG/HDL-C* triglyceride to high-density lipoprotein cholesterol ratio.
