# Supplementary material for: Incremental value of insulin resistance surrogate indices beyond the CHA2DS2-VASc score for predicting thromboembolic events in patients with chronic heart failure and atrial fibrillation
Source: Front Endocrinol (Lausanne). 2026 Sep 23;17:1954348. doi: 10.3389/fendo.2026.1954348 (PMC13641996; doi:10.3389/fendo.2026.1954348)
Supplement: Supplementary file 2 [file Table1.docx]

| **Table S1** Univariable Cox regression analysis of factors associated with IS/SE | | |
| --- | --- | --- |
| **Characteristics** | **Unadjusted HR (95% CI)** | ***P* value** |
| Age | 1.01 (1.00-1.02) | 0.003 |
| Male | 0.73 (0.59-0.90) | 0.003 |
| HR | 1.00 (0.99-1.00) | 0.557 |
| BMI | 1.08 (1.06-1.11) | <0.001 |
| MAP, per SD | 1.02 (0.92-1.13) | 0.741 |
| Current/ex-Smoker | 0.76 (0.60-0.96) | 0.024 |
| NYHA functional class III | 1.02 (0.81-1.29) | 0.852 |
| NYHA functional class IV | 0.98 (0.74-1.31) | 0.909 |
| Chronic kidney disease | 1.12 (0.90-1.41) | 0.317 |
| Hypertension | 1.30 (1.04-1.63) | 0.020 |
| Diabetes | 1.31 (1.06-1.62) | 0.012 |
| Stroke | 1.31 (1.04-1.64) | 0.022 |
| PAD | 1.27 (0.94-1.72) | 0.112 |
| Ischemic etiology | 1.04 (0.83-1.31) | 0.712 |
| White blood cell, per SD | 1.14 (1.04-1.25) | 0.006 |
| Hemoglobin, per SD | 0.96 (0.87-1.07) | 0.475 |
| Platelets, per SD | 0.97 (0.87-1.09) | 0.628 |
| ALT, per SD | 1.05 (0.99-1.11) | 0.098 |
| AST, per SD | 0.99 (0.86-1.15) | 0.945 |
| Creatinine, per SD | 0.90 (0.79-1.02) | 0.103 |
| eGFR, per SD | 0.99 (0.89-1.11) | 0.889 |
| FBG, per SD | 1.14 (1.04-1.25) | 0.006 |
| TG, per SD | 1.19 (1.12-1.28) | <0.001 |
| TC, per SD | 1.04 (0.94-1.16) | 0.440 |
| LDL-C, per SD | 1.00 (0.90-1.12) | 0.941 |
| HDL-C, per SD | 0.81 (0.72-0.92) | <0.001 |
| Potassium, per SD | 1.02 (0.94-1.12) | 0.618 |
| NT-proBNP, per SD | 1.10 (1.01-1.21) | 0.033 |
| LVEF, per SD | 1.04 (0.94-1.15) | 0.484 |
| Antiplatelet agents | 0.75 (0.61-0.93) | 0.010 |
| ACEIs/ARBs/ARNIs | 1.09 (0.88-1.35) | 0.428 |
| Beta-blocker | 1.09 (0.86-1.36) | 0.478 |
| CCB | 1.08 (0.81-1.43) | 0.600 |
| Statins | 1.13 (0.92-1.40) | 0.246 |
| DOACs | 0.76 (0.61-0.95) | 0.014 |
| Warfarin | 0.99 (0.75-1.30) | 0.940 |
| Digoxin | 1.03 (0.82-1.30) | 0.784 |
| MRAs | 0.94 (0.74-1.18) | 0.566 |
| Diuretics | 0.93 (0.75-1.16) | 0.526 |
| SGLT2 inhibitors | 1.02 (0.70-1.49) | 0.919 |
| Insulin | 1.01 (0.66-1.55) | 0.949 |
| Other oral antidiabetic agents | 1.06 (0.83-1.37) | 0.633 |

| **Table S2** Baseline characteristics of patients with CHF and AF according to tertiles of the TyG index | | | | |
| --- | --- | --- | --- | --- |
| **Variables** | **T1 (n=1491)** | **T2 (n=1491)** | **T3 (n=1492)** | ***P* value** |
| TyG index | ＜8.41 | 8.41-8.94 | ≥ 8.94 | - |
| Demographics |  |  |  |  |
| Age (years) | 68.0 (58.0-77.0) | 66.0 (57.0-75.0) | 64.0 (55.0-74.0) | <0.001 |
| Male (%) | 945 (63.4%) | 885 (59.4%) | 855 (57.3%) | 0.003 |
| Current/ex-Smoker (%) | 494 (33.1%) | 453 (30.4%) | 470 (31.5%) | 0.268 |
| Medical measurements |  |  |  |  |
| BMI (kg/m^2^) | 24.5 (22.2-27.3) | 24.9 (22.5-27.4) | 25.3 (23.0-27.8) | <0.001 |
| MAP (mmHg) | 93.3 (84.7-102.5) | 95.3 (86.3-104.3) | 97.0 (87.7-105.3) | <0.001 |
| HR (bpm) | 78.0 (68.0-89.0) | 78.0 (66.0-89.0) | 77.0 (68.0-88.0) | 0.726 |
| NYHA classification (%) |  |  |  | 0.965 |
| I-II | 586 (39.3%) | 601 (40.3%) | 589 (39.5%) |  |
| III | 586 (39.3%) | 582 (39.0%) | 581 (38.9%) |  |
| IV | 319 (21.4%) | 308 (20.7%) | 322 (21.6%) |  |
| Medical history (%) |  |  |  |  |
| CKD | 459 (30.8%) | 475 (31.9%) | 451 (30.2%) | 0.619 |
| Hypertension | 876 (58.8%) | 940 (63.0%) | 963 (64.5%) | 0.003 |
| Diabetes | 343 (23.0%) | 558 (37.4%) | 922 (61.8%) | <0.001 |
| Stroke | 383 (25.7%) | 378 (25.4%) | 382 (25.6%) | 0.976 |
| PAD | 191 (12.8%) | 173 (11.6%) | 181 (12.1%) | 0.600 |
| Ischemic etiology | 443 (29.7%) | 483 (32.4%) | 485 (32.5%) | 0.177 |
| Paroxysmal AF | 420 (28.2%) | 421 (28.2%) | 410 (27.5%) | 0.878 |
| CHA₂DS₂-VASc score | 4.02 ± 1.71 | 4.20 ± 1.75 | 4.37 ± 1.78 | <0.001 |
| Laboratory measurements |  |  |  |  |
| WBC (10^9^/L) | 6.02 (4.85-7.56) | 6.51 (5.24-8.02) | 6.65 (5.37-8.28) | <0.001 |
| Hemoglobin (g/L) | 128.0 (113.0-141.0) | 131.0 (117.3-143.5) | 132.0 (119.2-145.0) | <0.001 |
| Platelets (10^9^/L) | 185.0 (147.0-232.0) | 199.0 (162.0-243.5) | 209.0 (171.0-252.0) | <0.001 |
| ALT (U/L) | 22.0 (15.4-34.0) | 23.0 (16.0-35.0) | 24.0 (17.0-37.0) | <0.001 |
| AST (U/L) | 22.5 (17.0-31.0) | 22.1 (17.0-32.0) | 22.0 (17.3-31.0) | 0.886 |
| Creatinine (µmol/L) | 76.9 (65.0-91.0) | 76.3 (64.2-92.0) | 75.0 (62.0-88.5) | 0.006 |
| eGFR (mL/min/1.73m^2^) | 83.2 (65.9-95.2) | 83.3 (65.5-96.3) | 86.1 (68.4-98.5) | <0.001 |
| FBG (mmol/L) | 4.94 (4.36-5.59) | 5.52 (4.86-6.51) | 6.81 (5.58-9.14) | <0.001 |
| TG (mmol/L) | 0.80 (0.63-0.97) | 1.30 (1.07-1.54) | 2.00 (1.62-2.64) | <0.001 |
| TC (mmol/L) | 3.53 (2.96-4.22) | 3.96 (3.34-4.61) | 4.26 (3.58-5.08) | <0.001 |
| LDL-C (mmol/L) | 2.02 (1.55-2.60) | 2.37 (1.84-2.91) | 2.45 (1.89-3.03) | <0.001 |
| HDL-C (mmol/L) | 1.14 (0.95-1.38) | 1.08 (0.91-1.29) | 0.96 (0.80-1.15) | <0.001 |
| Potassium (mmol/L) | 3.96 (3.68-4.29) | 3.99 (3.72-4.28) | 3.92 (3.65-4.23) | <0.001 |
| NT-proBNP (pg/mL) | 2024.00 (1083.0-4019.0) | 1946.0 (1069.5-3740.0) | 2011.5 (1051.5-4029.0) | 0.619 |
| Echocardiography |  |  |  |  |
| LVEF (%) | 49.0 (41.0-57.0) | 51.0 (41.0-58.0) | 50.0 (41.0-57.0) | 0.092 |
| Medications at discharge (%) |  |  |  |  |
| Antiplatelet agents | 615 (41.2%) | 671 (45.0%) | 727 (48.7%) | <0.001 |

| **Table S2** (continued) | | | | |
| --- | --- | --- | --- | --- |
| **Variables** | **T1 (n=1491)** | **T2 (n=1491)** | **T3 (n=1492)** | ***P* value** |
| ACEIs/ARBs/ARNIs | 641 (43.0%) | 630 (42.3%) | 651 (43.6%) | 0.748 |
| Beta-blocker | 985 (66.1%) | 1008 (67.6%) | 1050 (70.4%) | 0.038 |
| CCB | 208 (14.0%) | 241 (16.2%) | 249 (16.7%) | 0.091 |
| Statins | 759 (50.9%) | 798 (53.5%) | 876 (58.7%) | <0.001 |
| DOACs | 1074 (72.0%) | 1088 (73.0%) | 1085 (72.7%) | 0.838 |
| Warfarin | 223 (15.0%) | 217 (14.6%) | 234 (15.7%) | 0.682 |
| Digoxin | 455 (30.5%) | 454 (30.4%) | 446 (29.9%) | 0.921 |
| MRAs | 1076 (72.2%) | 1079 (72.4%) | 1055 (70.7%) | 0.548 |
| Diuretics | 948 (63.6%) | 941 (63.1%) | 981 (65.8%) | 0.277 |
| SGLT2 inhibitors | 77 (5.2%) | 154 (10.3%) | 226 (15.1%) | <0.001 |
| Insulin | 65 (4.4%) | 87 (5.8%) | 179 (12.0%) | <0.001 |
| Other oral antidiabetic agents | 202 (13.5%) | 287 (19.2%) | 499 (33.4%) | <0.001 |
| Clinical outcome: IS/SE |  |  |  |  |
| Incidence/1000 person-years | 12.73 | 18.59 | 24.47 | <0.001 |

*CHF* chronic heart failure, *AF* atrial fibrillation, *TyG* triglyceride-glucose, *BMI* body mass index, *MAP* mean arterial pressure, *HR* heart rate, *NYHA* New York Heart Association, *CKD* chronic kidney disease, *PAD* peripheral arterial disease, *PCI* percutaneous coronary intervention, *CABG* coronary artery bypass grafting, *WBC* white blood cell, *AST* aspartate aminotransferase, *ALT* alanine aminotransferase, *eGFR* estimated glomerular filtration rate, *FBG* fasting blood glucose, *TG* triglyceride, *TC* total cholesterol, *LDL-C* low-density lipoprotein cholesterol, *HDL-C* high-density lipoprotein cholesterol, *NT-proBNP* N-terminal pro-B-type natriuretic peptide, *LVEF* left ventricular ejection fraction, *ACEI* angiotensin-converting enzyme inhibitor, *ARB* angiotensin receptor blocker, *ARNI* angiotensin receptor-neprilysin inhibitor, *CCB* calcium channel blocker, *DOACs* direct oral anticoagulants, *MRAs* mineralocorticoid receptor antagonists, *SGLT2 inhibitors* sodium-glucose cotransporter 2 inhibitors, *IS/SE* ischemic stroke/systemic embolism. Ischemic etiology was defined as a history of myocardial infarction, prior PCI, or prior CABG.

| **Table S3** Baseline characteristics of patients with CHF and AF according to tertiles of the TyG-BMI index | | | | |
| --- | --- | --- | --- | --- |
| **Variables** | **T1 (n=1491)** | **T2 (n=1491)** | **T3 (n=1492)** | ***P* value** |
| TyG-BMI index | ＜200.7 | 200.7-233.5 | ≥ 233.5 | - |
| Demographics |  |  |  |  |
| Age (years) | 67.0 (57.0-76.0) | 66.0 (57.0-75.0) | 65.0 (55.0-75.0) | 0.018 |
| Male (%) | 908 (60.9%) | 928 (62.2%) | 849 (56.9%) | 0.008 |
| Current/ex-Smoker (%) | 481 (32.3%) | 467 (31.3%) | 469 (31.4%) | 0.834 |
| Medical measurements |  |  |  |  |
| BMI (kg/m^2^) | 21.6 (20.0-23.0) | 24.9 (23.9-26.2) | 28.5 (27.0-30.7) | <0.001 |
| MAP (mmHg) | 93.0 ± 13.3 | 95.2 ± 13.5 | 97.2 ± 13.0 | <0.001 |
| HR (bpm) | 78.0 (67.0-89.0) | 78.0 (68.0-88.0) | 78.0 (67.0-88.0) | 0.813 |
| NYHA classification (%) |  |  |  | 0.005 |
| I-II | 566 (38.0%) | 587 (39.4%) | 623 (41.8%) |  |
| III | 562 (37.7%) | 603 (40.4%) | 584 (39.1%) |  |
| IV | 363 (24.3%) | 301 (20.2%) | 285 (19.1%) |  |
| Medical history (%) |  |  |  |  |
| CKD | 443 (29.7%) | 477 (32.0%) | 465 (31.2%) | 0.395 |
| Hypertension | 828 (55.5%) | 945 (63.4%) | 1006 (67.4%) | <0.001 |
| Diabetes | 443 (29.7%) | 602 (40.4%) | 778 (52.1%) | <0.001 |
| Stroke | 390 (26.2%) | 364 (24.4%) | 389 (26.1%) | 0.469 |
| PAD | 184 (12.3%) | 172 (11.5%) | 189 (12.7%) | 0.623 |
| Ischemic etiology | 468 (31.4%) | 457 (30.7%) | 486 (32.6%) | 0.522 |
| Paroxysmal AF | 462 (31.0%) | 380 (25.5%) | 409 (27.4%) | 0.003 |
| CHA₂DS₂-VASc score | 4.06 ± 1.80 | 4.14 ± 1.69 | 4.39 ± 1.75 | <0.001 |
| Laboratory measurements |  |  |  |  |
| WBC (10^9^/L) | 6.28 (5.04-7.96) | 6.37 (5.11-7.93) | 6.55 (5.26-8.07) | 0.017 |
| Hemoglobin (g/L) | 129.0 (115.0-142.0) | 130.3 (118.0-143.4) | 132.0 (118.0-144.9) | <0.001 |
| Platelets (10^9^/L) | 192.0 (151.0-237.5) | 201.0 (161.0-246.0) | 203.5 (167.0-249.0) | <0.001 |
| ALT (U/L) | 22.6 (16.0-35.0) | 23.0 (16.0-34.0) | 23.9 (16.3-37.0) | 0.048 |
| AST (U/L) | 22.9 (17.2-32.0) | 22.0 (17.0-31.0) | 22.0 (17.5-31.0) | 0.138 |
| Creatinine (µmol/L) | 76.0 (63.8-90.0) | 76.0 (64.6-90.5) | 76.0 (63.7-91.0) | 0.947 |
| eGFR (mL/min/1.73m^2^) | 84.2 (66.2-96.7) | 84.4 (67.4-96.7) | 84.0 (66.2-96.3) | 0.927 |
| FBG (mmol/L) | 5.16 (4.51-6.15) | 5.60 (4.86-6.75) | 6.13 (5.06-7.94) | <0.001 |
| TG (mmol/L) | 0.98 (0.72-1.35) | 1.23 (0.90-1.73) | 1.62 (1.15-2.21) | <0.001 |
| TC (mmol/L) | 3.73 (3.09-4.48) | 3.93 (3.23-4.66) | 4.06 (3.43-4.81) | <0.001 |
| LDL-C (mmol/L) | 2.17 (1.65-2.76) | 2.31 (1.76-2.90) | 2.36 (1.82-2.93) | <0.001 |
| HDL-C (mmol/L) | 1.12 (0.94-1.34) | 1.07 (0.89-1.27) | 1.00 (0.82-1.22) | <0.001 |
| Potassium (mmol/L) | 3.98 (3.67-4.29) | 3.95 (3.70-4.28) | 3.94 (3.66-4.24) | 0.062 |
| NT-proBNP (pg/mL) | 2217.0 (1158.0-4426.5) | 1938.0 (1034.5-3685.5) | 1895.0 (1044.8-3688.5) | <0.001 |
| Echocardiography |  |  |  |  |
| LVEF (%) | 50.0 (40.0-57.0) | 50.0 (41.0-57.0) | 50.0 (41.0-57.0) | 0.991 |
| Medications at discharge (%) |  |  |  |  |
| Antiplatelet agents | 642 (43.1%) | 653 (43.8%) | 718 (48.1%) | 0.011 |

| **Table S3** (continued) | | | | |
| --- | --- | --- | --- | --- |
| **Variables** | **T1 (n=1491)** | **T2 (n=1491)** | **T3 (n=1492)** | ***P* value** |
| ACEIs/ARBs/ARNIs | 617 (41.4%) | 638 (42.8%) | 667 (44.7%) | 0.184 |
| Beta-blocker | 965 (64.7%) | 1009 (67.7%) | 1069 (71.6%) | <0.001 |
| CCB | 191 (12.8%) | 235 (15.8%) | 272 (18.2%) | <0.001 |
| Statins | 736 (49.4%) | 814 (54.6%) | 883 (59.2%) | <0.001 |
| DOACs | 1077 (72.2%) | 1109 (74.4%) | 1061 (71.1%) | 0.127 |
| Warfarin | 218 (14.6%) | 216 (14.5%) | 240 (16.1%) | 0.400 |
| Digoxin | 469 (31.5%) | 452 (30.3%) | 434 (29.1%) | 0.372 |
| MRAs | 1076 (72.2%) | 1061 (71.2%) | 1073 (71.9%) | 0.817 |
| Diuretics | 929 (62.3%) | 946 (63.4%) | 995 (66.7%) | 0.035 |
| SGLT2 inhibitors | 125 (8.4%) | 162 (10.9%) | 170 (11.4%) | 0.015 |
| Insulin | 69 (4.6%) | 111 (7.4%) | 151 (10.1%) | <0.001 |
| Other oral antidiabetic agents | 228 (15.3%) | 330 (22.1%) | 430 (28.8%) | <0.001 |
| Clinical outcome: IS/SE |  |  |  |  |
| Incidence/1000 person-years | 9.82 | 15.92 | 29.98 | <0.001 |

*CHF* chronic heart failure, *AF* atrial fibrillation, *TyG-BMI index* triglyceride-glucose body mass index, *BMI* body mass index, *MAP* mean arterial pressure, *HR* heart rate, *NYHA* New York Heart Association, *CKD* chronic kidney disease, *PAD* peripheral arterial disease, *PCI* percutaneous coronary intervention, *CABG* coronary artery bypass grafting, *WBC* white blood cell, *AST* aspartate aminotransferase, *ALT* alanine aminotransferase, *eGFR* estimated glomerular filtration rate, *FBG* fasting blood glucose, *TG* triglyceride, *TC* total cholesterol, *LDL-C* low-density lipoprotein cholesterol, *HDL-C* high-density lipoprotein cholesterol, *NT-proBNP* N-terminal pro-B-type natriuretic peptide, *LVEF* left ventricular ejection fraction, *ACEI* angiotensin-converting enzyme inhibitor, *ARB* angiotensin receptor blocker, *ARNI* angiotensin receptor-neprilysin inhibitor, *CCB* calcium channel blocker, *DOACs* direct oral anticoagulants, *MRAs* mineralocorticoid receptor antagonists, *SGLT2 inhibitors* sodium-glucose cotransporter 2 inhibitors, *IS/SE* ischemic stroke/systemic embolism. Ischemic etiology was defined as a history of myocardial infarction, prior PCI, or prior CABG.

| **Table S4** Baseline characteristics of patients with CHF and AF according to tertiles of METS-IR | | | | |
| --- | --- | --- | --- | --- |
| **Variables** | **T1 (n=1491)** | **T2 (n=1491)** | **T3 (n=1492)** | ***P* value** |
| METS-IR | ＜36.2 | 36.2-42.6 | ≥ 42.6 | - |
| Demographics |  |  |  |  |
| Age (years) | 67.0 (57.0-76.0) | 66.0 (57.0-75.0) | 65.0 (55.0-74.0) | 0.002 |
| Male (%) | 851 (57.1%) | 905 (60.7%) | 929 (62.3%) | 0.012 |
| Current/ex-Smoker (%) | 433 (29.0%) | 475 (31.9%) | 509 (34.1%) | 0.012 |
| Medical measurements |  |  |  |  |
| BMI (kg/m^2^) | 21.8 (20.0-23.4) | 25.0 (23.8-26.5) | 28.3 (26.5-30.6) | <0.001 |
| MAP (mmHg) | 93.3 (84.7-102.3) | 95.0 (86.7-104.3) | 96.7 (88.2-105.7) | <0.001 |
| HR (bpm) | 77.0 (67.0-88.0) | 78.0 (67.0-88.0) | 78.0 (68.0-90.0) | 0.073 |
| NYHA classification (%) |  |  |  | 0.101 |
| I-II | 586 (39.3%) | 594 (39.8%) | 596 (39.9%) |  |
| III | 555 (37.2%) | 592 (39.7%) | 602 (40.3%) |  |
| IV | 350 (23.5%) | 305 (20.5%) | 294 (19.7%) |  |
| Medical history (%) |  |  |  |  |
| CKD | 454 (30.4%) | 449 (30.1%) | 482 (32.3%) | 0.378 |
| Hypertension | 846 (56.7%) | 918 (61.6%) | 1015 (68.0%) | <0.001 |
| Diabetes | 429 (28.8%) | 619 (41.5%) | 775 (51.9%) | <0.001 |
| Stroke | 399 (26.8%) | 355 (23.8%) | 389 (26.1%) | 0.154 |
| PAD | 196 (13.1%) | 165 (11.1%) | 184 (12.3%) | 0.217 |
| Ischemic etiology | 452 (30.3%) | 470 (31.5%) | 489 (32.8%) | 0.352 |
| Paroxysmal AF | 455 (30.5%) | 391 (26.2%) | 405 (27.1%) | 0.023 |
| CHA₂DS₂-VASc score | 4.11 ± 1.79 | 4.16 ± 1.71 | 4.32 ± 1.75 | 0.002 |
| Laboratory measurements |  |  |  |  |
| WBC (10^9^/L) | 6.17 (5.02-7.80) | 6.38 (5.09-7.92) | 6.59 (5.32-8.16) | <0.001 |
| Hemoglobin (g/L) | 129.0 (115.0-142.0) | 130.0 (117.4-143.6) | 132.0 (117.9-145.0) | <0.001 |
| Platelets (10^9^/L) | 195.0 (153.0-239.0) | 199.0 (161.0-245.0) | 202.0 (165.0-247.0) | <0.001 |
| ALT (U/L) | 22.7 (16.0-35.0) | 23.0 (16.0-35.0) | 23.7 (16.0-37.0) | 0.114 |
| AST (U/L) | 22.8 (17.7-32.0) | 22.0 (17.0-31.0) | 22.0 (17.3-31.0) | 0.307 |
| Creatinine (µmol/L) | 75.0 (63.0-89.3) | 76.0 (65.0-89.0) | 77.0 (64.8-94.6) | 0.012 |
| eGFR (mL/min/1.73m^2^) | 84.5 (66.5-97.0) | 84.4 (68.4-96.2) | 83.5 (64.9-96.7) | 0.614 |
| FBG (mmol/L) | 5.14 (4.52-6.12) | 5.61 (4.86-6.80) | 6.12 (5.05-7.90) | <0.001 |
| TG (mmol/L) | 1.03 (0.75-1.40) | 1.26 (0.90-1.78) | 1.54 (1.05-2.17) | <0.001 |
| TC (mmol/L) | 3.94 (3.29-4.72) | 3.93 (3.25-4.64) | 3.88 (3.19-4.61) | 0.188 |
| LDL-C (mmol/L) | 2.25 (1.71-2.87) | 2.31 (1.77-2.87) | 2.27 (1.75-2.86) | 0.333 |
| HDL-C (mmol/L) | 1.24 (1.05-1.47) | 1.07 (0.91-1.25) | 0.89 (0.75-1.07) | <0.001 |
| Potassium (mmol/L) | 3.99 (3.69-4.29) | 3.94 (3.68-4.27) | 3.94 (3.66-4.25) | 0.086 |
| NT-proBNP (pg/mL) | 2056.0 (1126.0-4165.0) | 1997.0 (1051.0-3854.5) | 1921.0 (1050.8-3789.0) | 0.166 |
| Echocardiography |  |  |  |  |
| LVEF (%) | 50.0 (41.0-57.0) | 50.0 (41.0-57.0) | 50.0 (41.0-57.0) | 0.834 |
| Medications at discharge (%) |  |  |  |  |
| Antiplatelet agents | 635 (42.6%) | 668 (44.8%) | 710 (47.6%) | 0.023 |

| **Table S4** (continued) | | | | |
| --- | --- | --- | --- | --- |
| **Variables** | **T1 (n=1491)** | **T2 (n=1491)** | **T3 (n=1492)** | ***P* value** |
| ACEIs/ARBs/ARNIs | 620 (41.6%) | 640 (42.9%) | 662 (44.4%) | 0.306 |
| Beta-blocker | 966 (64.8%) | 1003 (67.3%) | 1074 (72.0%) | <0.001 |
| CCB | 189 (12.7%) | 239 (16.0%) | 270 (18.1%) | <0.001 |
| Statins | 745 (50.0%) | 818 (54.9%) | 870 (58.3%) | <0.001 |
| DOACs | 1121 (75.2%) | 1065 (71.4%) | 1061 (71.1%) | 0.021 |
| Warfarin | 199 (13.3%) | 236 (15.8%) | 239 (16.0%) | 0.075 |
| Digoxin | 443 (29.7%) | 478 (32.1%) | 434 (29.1%) | 0.177 |
| MRAs | 1086 (72.8%) | 1052 (70.6%) | 1072 (71.8%) | 0.382 |
| Diuretics | 935 (62.7%) | 949 (63.6%) | 986 (66.1%) | 0.140 |
| SGLT2 inhibitors | 131 (8.8%) | 158 (10.6%) | 168 (11.3%) | 0.069 |
| Insulin | 64 (4.3%) | 116 (7.8%) | 151 (10.1%) | <0.001 |
| Other oral antidiabetic agents | 219 (14.7%) | 344 (23.1%) | 425 (28.5%) | <0.001 |
| Clinical outcome: IS/SE |  |  |  |  |
| Incidence/1000 person-years | 9.10 | 15.78 | 30.79 | <0.001 |

*CHF* chronic heart failure, *AF* atrial fibrillation, *METS-IR* metabolic score for insulin resistance, *BMI* body mass index, *MAP* mean arterial pressure, *HR* heart rate, *NYHA* New York Heart Association, *CKD* chronic kidney disease, *PAD* peripheral arterial disease, *PCI* percutaneous coronary intervention, *CABG* coronary artery bypass grafting, *WBC* white blood cell, *AST* aspartate aminotransferase, *ALT* alanine aminotransferase, *eGFR* estimated glomerular filtration rate, *FBG* fasting blood glucose, *TG* triglyceride, *TC* total cholesterol, *LDL-C* low-density lipoprotein cholesterol, *HDL-C* high-density lipoprotein cholesterol, *NT-proBNP* N-terminal pro-B-type natriuretic peptide, *LVEF* left ventricular ejection fraction, *ACEI* angiotensin-converting enzyme inhibitor, *ARB* angiotensin receptor blocker, *ARNI* angiotensin receptor-neprilysin inhibitor, *CCB* calcium channel blocker, *DOACs* direct oral anticoagulants, *MRAs* mineralocorticoid receptor antagonists, *SGLT2 inhibitors* sodium-glucose cotransporter 2 inhibitors, *IS/SE* ischemic stroke/systemic embolism. Ischemic etiology was defined as a history of myocardial infarction, prior PCI, or prior CABG.

| **Table S5** Baseline characteristics of patients with CHF and AF according to tertiles of the TG/HDL-C ratio | | | | |
| --- | --- | --- | --- | --- |
| **Variables** | **T1 (n=1491)** | **T2 (n=1491)** | **T3 (n=1492)** | ***P* value** |
| TG/HDL-C | ＜0.89 | 0.89-1.56 | ≥1.56 | - |
| Demographics |  |  |  |  |
| Age (years) | 69.0 (59.0-77.0) | 66.0 (57.0-75.0) | 63.0 (54.0-73.0) | <0.001 |
| Male (%) | 849 (56.9%) | 902 (60.5%) | 934 (62.6%) | 0.006 |
| Current/ex-Smoker (%) | 451 (30.2%) | 447 (30.0%) | 519 (34.8%) | 0.007 |
| Medical measurements |  |  |  |  |
| BMI (kg/m^2^) | 24.5 (22.1-27.2) | 25.0 (22.5-27.6) | 25.2 (22.9-27.7) | <0.001 |
| MAP (mmHg) | 93.7 (85.0-103.3) | 95.0 (86.7-104.3) | 96.7 (87.3-105.3) | <0.001 |
| HR (bpm) | 77.0 (66.0-88.0) | 78.0 (67.0-88.0) | 78.0 (68.0-90.0) | 0.148 |
| NYHA classification (%) |  |  |  | 0.916 |
| I-II | 581 (39.0%) | 593 (39.8%) | 602 (40.3%) |  |
| III | 590 (39.6%) | 576 (38.6%) | 583 (39.1%) |  |
| IV | 320 (21.5%) | 322 (21.6%) | 307 (20.6%) |  |
| Medical history (%) |  |  |  |  |
| CKD | 479 (32.1%) | 446 (29.9%) | 460 (30.8%) | 0.422 |
| Hypertension | 915 (61.4%) | 934 (62.6%) | 930 (62.3%) | 0.756 |
| Diabetes | 570 (38.2%) | 579 (38.8%) | 674 (45.2%) | <0.001 |
| Stroke | 404 (27.1%) | 354 (23.7%) | 385 (25.8%) | 0.106 |
| PAD | 199 (13.3%) | 180 (12.1%) | 166 (11.1%) | 0.177 |
| Ischemic etiology | 452 (30.3%) | 496 (33.3%) | 463 (31.0%) | 0.195 |
| Paroxysmal AF | 395 (26.5%) | 430 (28.8%) | 426 (28.6%) | 0.297 |
| CHA₂DS₂-VASc score | 4.35 ± 1.71 | 4.15 ± 1.77 | 4.09 ± 1.76 | <0.001 |
| Laboratory measurements |  |  |  |  |
| WBC (10^9^/L) | 5.93 (4.75-7.57) | 6.48 (5.25-7.90) | 6.71 (5.47-8.42) | <0.001 |
| Hemoglobin (g/L) | 126.0 (112.0-140.0) | 131.0 (117.4-144.0) | 133.3 (120.0-146.0) | <0.001 |
| Platelets (10^9^/L) | 187.0 (147.0-234.0) | 199.0 (161.5-247.0) | 208.5 (171.0-249.0) | <0.001 |
| ALT (U/L) | 21.8 (15.8-33.0) | 23.0 (16.0-35.3) | 25.0 (17.0-38.0) | <0.001 |
| AST (U/L) | 22.5 (17.4-31.2) | 22.1 (17.0-31.0) | 22.0 (17.0-31.4) | 0.590 |
| Creatinine (µmol/L) | 75.0 (63.7-89.9) | 76.4 (64.0-91.0) | 76.3 (64.5-92.0) | 0.362 |
| eGFR (mL/min/1.73m^2^) | 83.2 (65.9-95.1) | 84.0 (66.3-95.9) | 85.8 (67.9-99.2) | 0.001 |
| FBG (mmol/L) | 5.44 (4.69-6.75) | 5.53 (4.77-6.79) | 5.72 (4.90-7.06) | <0.001 |
| TG (mmol/L) | 0.78 (0.62-0.95) | 1.26 (1.05-1.48) | 2.01 (1.68-2.64) | <0.001 |
| TC (mmol/L) | 3.74 (3.06-4.49) | 3.91 (3.26-4.61) | 4.08 (3.43-4.88) | <0.001 |
| LDL-C (mmol/L) | 2.08 (1.58-2.66) | 2.37 (1.83-2.93) | 2.42 (1.84-2.98) | <0.001 |
| HDL-C (mmol/L) | 1.29 (1.12-1.53) | 1.07 (0.92-1.23) | 0.87 (0.74-1.01) | <0.001 |
| Potassium (mmol/L) | 4.00 (3.70-4.30) | 3.94 (3.68-4.26) | 3.93 (3.65-4.23) | <0.001 |
| NT-proBNP (pg/mL) | 2024.0 (1107.5-4033.0) | 1996.0 (1052.5-3766.0) | 1951.5 (1052.8-3945.0) | 0.408 |
| Echocardiography |  |  |  |  |
| LVEF (%) | 50.0 (41.0-57.0) | 50.0 (41.0-57.0) | 50.0 (41.0-57.0) | 0.771 |
| Medications at discharge (%) |  |  |  |  |
| Antiplatelet agents | 641 (43.0%) | 683 (45.8%) | 689 (46.2%) | 0.160 |

| **Table S5** (continued) | | | | |
| --- | --- | --- | --- | --- |
| **Variables** | **T1 (n=1491)** | **T2 (n=1491)** | **T3 (n=1492)** | ***P* value** |
| ACEIs/ARBs/ARNIs | 648 (43.5%) | 629 (42.2%) | 645 (43.2%) | 0.755 |
| Beta-blocker | 983 (65.9%) | 1004 (67.3%) | 1056 (70.8%) | 0.014 |
| CCB | 201 (13.5%) | 239 (16.0%) | 258 (17.3%) | 0.014 |
| Statins | 792 (53.1%) | 798 (53.5%) | 843 (56.5%) | 0.128 |
| DOACs | 1083 (72.6%) | 1098 (73.6%) | 1066 (71.4%) | 0.405 |
| Warfarin | 220 (14.8%) | 212 (14.2%) | 242 (16.2%) | 0.286 |
| Digoxin | 445 (29.8%) | 447 (30.0%) | 463 (31.0%) | 0.742 |
| MRAs | 1053 (70.6%) | 1080 (72.4%) | 1077 (72.2%) | 0.492 |
| Diuretics | 929 (62.3%) | 972 (65.2%) | 969 (64.9%) | 0.191 |
| SGLT2 inhibitors | 138 (9.3%) | 153 (10.3%) | 166 (11.1%) | 0.240 |
| Insulin | 108 (7.2%) | 108 (7.2%) | 115 (7.7%) | 0.855 |
| Other oral antidiabetic agents | 319 (21.4%) | 314 (21.1%) | 355 (23.8%) | 0.146 |
| Clinical outcome: IS/SE |  |  |  |  |
| Incidence/1000 person-years | 12.38 | 18.65 | 24.29 | <0.001 |

*CHF* chronic heart failure, *AF* atrial fibrillation, *TG/HDL-C* triglyceride to high-density lipoprotein cholesterol ratio, *BMI* body mass index, *MAP* mean arterial pressure, *HR* heart rate, *NYHA* New York Heart Association, *CKD* chronic kidney disease, *PAD* peripheral arterial disease, *PCI* percutaneous coronary intervention, *CABG* coronary artery bypass grafting, *WBC* white blood cell, *AST* aspartate aminotransferase, *ALT* alanine aminotransferase, *eGFR* estimated glomerular filtration rate, *FBG* fasting blood glucose, *TG* triglyceride, *TC* total cholesterol, *LDL-C* low-density lipoprotein cholesterol, *HDL-C* high-density lipoprotein cholesterol, *NT-proBNP* N-terminal pro-B-type natriuretic peptide, *LVEF* left ventricular ejection fraction, *ACEI* angiotensin-converting enzyme inhibitor, *ARB* angiotensin receptor blocker, *ARNI* angiotensin receptor-neprilysin inhibitor, *CCB* calcium channel blocker, *DOACs* direct oral anticoagulants, *MRAs* mineralocorticoid receptor antagonists, *SGLT2 inhibitors* sodium-glucose cotransporter 2 inhibitors, *IS/SE* ischemic stroke/systemic embolism. Ischemic etiology was defined as a history of myocardial infarction, prior PCI, or prior CABG.

| **Table S6** Associations between IR surrogate indices and IS/SE accounting for all-cause death as a competing risk | | | | | | | | | | | | |
| --- | --- | --- | --- | --- | --- | --- | --- | --- | --- | --- | --- | --- |
| **Categories** | **Incidence/**  **1000 person-y** | **Unadjusted** |  |  |  | **Model 1** |  |  |  | **Model 2** |  |  |
|  |  | **sHR (95% CI)** | ***P*-value** | ***P* for trend** |  | **sHR (95% CI)** | ***P*-value** | ***P* for trend** |  | **sHR (95% CI)** | ***P*-value** | ***P* for trend** |
| TyG index |  |  |  |  |  |  |  |  |  |  |  |  |
| Per 1-SD increase |  | 1.29 (1.17-1.43) | <0.001 |  |  | 1.30 (1.18-1.44) | <0.001 |  |  | 1.27 (1.12-1.43) | <0.001 |  |
| Tertilesᵃ |  |  |  | <0.001 |  |  |  | <0.001 |  |  |  | 0.004 |
| T1 | 12.73 | Ref. |  |  |  | Ref. |  |  |  | Ref. |  |  |
| T2 | 18.59 | 1.47 (1.11-1.94) | 0.007 |  |  | 1.46 (1.11-1.93) | 0.007 |  |  | 1.39 (1.04-1.86) | 0.024 |  |
| T3 | 24.47 | 1.77 (1.36-2.32) | <0.001 |  |  | 1.78 (1.36-2.33) | <0.001 |  |  | 1.58 (1.16-2.16) | 0.004 |  |
| TyG-BMI index |  |  |  |  |  |  |  |  |  |  |  |  |
| Per 1-SD increase |  | 1.43 (1.32-1.55) | <0.001 |  |  | 1.43 (1.32-1.54) | <0.001 |  |  | 1.41 (1.29-1.55) | <0.001 |  |
| Tertilesᵃ |  |  |  | <0.001 |  |  |  | <0.001 |  |  |  | <0.001 |
| T1 | 9.82 | Ref. |  |  |  | Ref. |  |  |  | Ref. |  |  |
| T2 | 15.92 | 1.64 (1.20-2.24) | 0.002 |  |  | 1.65 (1.20-2.25) | 0.002 |  |  | 1.70 (1.23-2.35) | 0.001 |  |
| T3 | 29.98 | 2.97 (2.23-3.96) | <0.001 |  |  | 2.97 (2.23-3.97) | <0.001 |  |  | 2.98 (2.17-4.09) | <0.001 |  |
| METS-IR |  |  |  |  |  |  |  |  |  |  |  |  |
| Per 1-SD increase |  | 1.24 (1.14-1.34) | <0.001 |  |  | 1.23 (1.14-1.34) | <0.001 |  |  | 1.28 (1.18-1.39) | <0.001 |  |
| Tertilesᵃ |  |  |  | <0.001 |  |  |  | <0.001 |  |  |  | <0.001 |
| T1 | 9.10 | Ref. |  |  |  | Ref. |  |  |  | Ref. |  |  |
| T2 | 15.78 | 1.70 (1.23-2.34) | 0.001 |  |  | 1.72 (1.25-2.37) | <0.001 |  |  | 1.75 (1.26-2.43) | <0.001 |  |
| T3 | 30.79 | 3.25 (2.43-4.36) | <0.001 |  |  | 3.38 (2.52-4.53) | <0.001 |  |  | 3.56 (2.62-4.83) | <0.001 |  |
| TG/HDL-C |  |  |  |  |  |  |  |  |  |  |  |  |
| Per 1-SD increase |  | 1.11 (1.06-1.17) | <0.001 |  |  | 1.11 (1.05-1.17) | <0.001 |  |  | 1.14 (1.08-1.20) | <0.001 |  |
| Tertilesᵃ |  |  |  | <0.001 |  |  |  | <0.001 |  |  |  | <0.001 |
| T1 | 12.38 | Ref. |  |  |  | Ref. |  |  |  | Ref. |  |  |
| T2 | 18.65 | 1.53 (1.16-2.04) | 0.003 |  |  | 1.57 (1.18-2.08) | 0.002 |  |  | 1.61 (1.20-2.15) | 0.001 |  |
| **Table S6** (continued) | | | | | | | | | | | | |
| **Categories** | **Incidence/**  **1000 person-y** | **Unadjusted** |  |  |  | **Model 1** |  |  |  | **Model 2** |  |  |
|  |  | **sHR (95% CI)** | ***P*-value** | ***P* for trend** |  | **sHR (95% CI)** | ***P*-value** | ***P* for trend** |  | **sHR (95% CI)** | ***P*-value** | ***P* for trend** |
| T3 | 24.29 | 1.94 (1.48-2.54) | <0.001 |  |  | 2.03 (1.54-2.68) | <0.001 |  |  | 1.95 (1.46-2.61) | <0.001 |  |

*IR* insulin resistance, *IS/SE* ischemic stroke/systemic embolism, *TyG* triglyceride-glucose, *TyG-BMI* triglyceride-glucose body mass index, *METS-IR* metabolic score for insulin resistance, *TG/HDL-C* triglyceride to high-density lipoprotein cholesterol ratio, *sHR* subdistribution hazard ratio, *CI* confidence interval, *SD* standard deviation. Fine–Gray models treated all-cause death before IS/SE as a competing event; estimates are presented as subdistribution hazard ratios (sHRs).

Model 1: adjusted for age, sex, and smoking status.

Model 2: adjusted for Model 1 plus MAP, heart rate, NYHA class, LVEF, NT-proBNP, LDL-C, WBC count, ALT, serum creatinine, diabetes mellitus, hypertension, prior stroke, PAD, ischemic etiology, anticoagulants, antiplatelet agents, statins, ACEIs/ARBs/ARNIs, beta-blockers, MRAs, digoxin, SGLT2 inhibitors, other glucose-lowering medications, and, where applicable, BMI, FBG, TG, and HDL-C (excluding components of the corresponding IR index).

ᵃ Tertiles were defined according to the distribution of each IR surrogate index.

| **Table S7** Associations between IR surrogate indices and IS/SE stratified by AF type | | | | | | | | | | |
| --- | --- | --- | --- | --- | --- | --- | --- | --- | --- | --- |
| **Subgroups** | **Incidence/**  **1000 person-y** | **Model 1** | | |  | **Model 2** | | |  | ***P* for**  **interaction** |
|  |  | **HR (95% CI)** | ***P*-value** | ***P* for trend** |  | **HR (95% CI)** | ***P*-value** | ***P* for trend** |  |  |
| TyG index |  |  |  |  |  |  |  |  |  |  |
| Paroxysmal AF |  |  |  | 0.001 |  |  |  | 0.074 |  | 0.954^a^ |
| T1 | 10.10 | Ref. |  |  |  | Ref. |  |  |  |  |
| T2 | 15.80 | 1.57 (0.88-2.79) | 0.129 |  |  | 1.41 (0.77-2.60) | 0.269 |  |  |  |
| T3 | 23.95 | 2.43 (1.41-4.20) | 0.001 |  |  | 1.79 (0.95-3.40) | 0.073 |  |  |  |
| Non-paroxysmal AF |  |  |  | <0.001 |  |  |  | 0.007 |  |  |
| T1 | 13.76 | Ref. |  |  |  | Ref. |  |  |  |  |
| T2 | 19.45 | 1.41 (1.03-1.94) | 0.034 |  |  | 1.35 (0.97-1.88) | 0.076 |  |  |  |
| T3 | 24.99 | 1.84 (1.35-2.52) | <0.001 |  |  | 1.63 (1.14-2.31) | 0.007 |  |  |  |
| TyG-BMI index |  |  |  |  |  |  |  |  |  |  |
| Paroxysmal AF |  |  |  | <0.001 |  |  |  | 0.003 |  | 0.672^b^ |
| T1 | 8.58 | Ref. |  |  |  | Ref. |  |  |  |  |
| T2 | 14.68 | 1.71 (0.92-3.19) | 0.088 |  |  | 1.63 (0.85-3.14) | 0.142 |  |  |  |
| T3 | 26.66 | 3.08 (1.74-5.46) | <0.001 |  |  | 2.50 (1.35-4.65) | 0.004 |  |  |  |
| Non-paroxysmal AF |  |  |  | <0.001 |  |  |  | <0.001 |  |  |
| T1 | 10.77 | Ref. |  |  |  | Ref. |  |  |  |  |
| T2 | 15.55 | 1.45 (1.01-2.08) | 0.047 |  |  | 1.55 (1.07-2.24) | 0.021 |  |  |  |
| T3 | 31.71 | 2.98 (2.15-4.13) | <0.001 |  |  | 3.14 (2.23-4.43) | <0.001 |  |  |  |
| METS-IR |  |  |  |  |  |  |  |  |  |  |
| Paroxysmal AF |  |  |  | <0.001 |  |  |  | <0.001 |  | 0.051^c^ |
| T1 | 7.53 | Ref. |  |  |  | Ref. |  |  |  |  |
| T2 | 15.11 | 1.99 (1.05-3.79) | 0.035 |  |  | 1.73 (0.89-3.35) | 0.107 |  |  |  |
| T3 | 27.39 | 3.75 (2.06-6.81) | <0.001 |  |  | 3.18 (1.71-5.93) | <0.001 |  |  |  |
| Non-paroxysmal AF |  |  |  | <0.001 |  |  |  | <0.001 |  |  |
| T1 | 9.91 | Ref. |  |  |  | Ref. |  |  |  |  |
| T2 | 16.50 | 1.70 (1.17-2.45) | 0.005 |  |  | 1.83 (1.26-2.65) | 0.002 |  |  |  |
| T3 | 31.56 | 3.35 (2.40-4.68) | <0.001 |  |  | 3.72 (2.63-5.26) | <0.001 |  |  |  |
| TG/HDL-C |  |  |  |  |  |  |  |  |  |  |
| Paroxysmal AF |  |  |  | <0.001 |  |  |  | 0.002 |  | 0.147^d^ |
| T1 | 10.36 | Ref. |  |  |  | Ref. |  |  |  |  |
| T2 | 15.22 | 1.51 (0.84-2.71) | 0.166 |  |  | 1.71 (0.92-3.17) | 0.091 |  |  |  |
| T3 | 23.92 | 2.54 (1.47-4.37) | <0.001 |  |  | 2.52 (1.41-4.50) | 0.002 |  |  |  |
| Non-paroxysmal AF |  |  |  | <0.001 |  |  |  | <0.001 |  |  |
| T1 | 12.64 | Ref. |  |  |  | Ref. |  |  |  |  |
| T2 | 20.64 | 1.67 (1.20-2.31) | 0.002 |  |  | 1.68 (1.20-2.33) | 0.002 |  |  |  |
| T3 | 24.43 | 2.01 (1.46-2.77) | <0.001 |  |  | 1.90 (1.37-2.64) | <0.001 |  |  |  |

*IR* insulin resistance, *IS/SE* ischemic stroke/systemic embolism, *AF* atrial fibrillation, *TyG* triglyceride-glucose, *TyG-BMI* triglyceride-glucose body mass index, *METS-IR* metabolic score for insulin resistance, *TG/HDL-C* triglyceride to high-density lipoprotein cholesterol ratio, *HR* hazard ratio, *CI* confidence interval.

Within each AF-type subgroup, tertiles were defined according to the subgroup-specific distribution of each IR surrogate index, with T1 as the reference.

Covariate adjustments for Models 1 and 2 were identical to those specified in Table 2 of the main manuscript.

^a-d^ *P* for interaction was derived from the interaction term between each standardized IR surrogate index and AF type in Model 2, with IS/SE as the endpoint.

| **Table S8** Incremental predictive value of IR surrogate indices beyond the CHA₂DS₂-VASc score for IS/SE stratified by AF type | | | | | | |
| --- | --- | --- | --- | --- | --- | --- |
|  | **AUC (95% CI)** | ***P* value** | **NRI (95% CI)** | ***P* value** | **IDI (95% CI)** | ***P* value** |
| Paroxysmal AF |  |  |  |  |  |  |
| CHA₂DS₂-VASc score | 0.687 (0.622-0.752) | Ref. | **-** | Ref. | **-** | Ref. |
| + TyG index | 0.692 (0.626-0.758) | 0.788 | 0.220 (0.019-0.488) | 0.030 | 0.005 (-0.001-0.021) | 0.106 |
| + TyG-BMI index | 0.707 (0.645-0.769) | 0.273 | 0.350 (0.090-0.582) | 0.016 | 0.010 (0.001-0.030) | 0.006 |
| + METS-IR | 0.716 (0.655-0.777) | 0.145 | 0.389 (0.119-0.618) | 0.002 | 0.012 (0.002-0.030) | 0.010 |
| + TG/HDL-C | 0.706 (0.642-0.771) | 0.194 | 0.268 (0.022-0.550) | 0.042 | 0.007 (0.000-0.035) | 0.046 |
| Pairwise comparison of AUC |  |  |  |  |  |  |
| + TyG index vs + TyG-BMI index | 0.692 vs 0.707 | 0.509 | **-** | **-** | **-** | **-** |
| + TyG index vs + METS-IR | 0.692 vs 0.716 | 0.322 | **-** | **-** | **-** | **-** |
| + TyG index vs + TG/HDL-C | 0.692 vs 0.706 | 0.376 | **-** | **-** | **-** | **-** |
| + TyG-BMI index vs + METS-IR | 0.707 vs 0.716 | 0.456 | **-** | **-** | **-** | **-** |
| + TyG-BMI index vs + TG/HDL-C | 0.707 vs 0.706 | 0.991 | **-** | **-** | **-** | **-** |
| + METS-IR vs + TG/HDL-C | 0.716 vs 0.706 | 0.614 | **-** | **-** | **-** | **-** |
| Non-paroxysmal AF |  |  |  |  |  |  |
| CHA₂DS₂-VASc score | 0.617 (0.575-0.659) | Ref. | **-** | Ref. | **-** | Ref. |
| + TyG index | 0.637 (0.595-0.679) | 0.263 | 0.201 (0.047-0.374) | 0.016 | 0.006 (0.002-0.015) | <0.001 |
| + TyG-BMI index | 0.661 (0.621-0.700) | 0.038 | 0.406 (0.213-0.555) | <0.001 | 0.010 (0.004-0.020) | <0.001 |
| + METS-IR | 0.672 (0.634-0.711) | <0.001 | 0.419 (0.227-0.559) | 0.020 | 0.004 (0.001-0.007) | 0.020 |
| + TG/HDL-C | 0.635 (0.593-0.677) | 0.002 | 0.143 (-0.088-0.301) | 0.094 | 0.001 (0.000-0.003) | 0.068 |
| Pairwise comparison of AUC |  |  |  |  |  |  |
| + TyG index vs + TyG-BMI index | 0.637 vs 0.661 | 0.205 | **-** | **-** | **-** | **-** |
| + TyG index vs + METS-IR | 0.637 vs 0.672 | 0.039 | **-** | **-** | **-** | **-** |
| + TyG index vs + TG/HDL-C | 0.637 vs 0.635 | 0.876 | **-** | **-** | **-** | **-** |
| + TyG-BMI index vs + METS-IR | 0.661 vs 0.672 | 0.344 | **-** | **-** | **-** | **-** |
| + TyG-BMI index vs + TG/HDL-C | 0.661 vs 0.635 | 0.208 | **-** | **-** | **-** | **-** |
| + METS-IR vs + TG/HDL-C | 0.672 vs 0.635 | 0.002 | **-** | **-** | **-** | **-** |

*IR* insulin resistance, *IS/SE* ischemic stroke/systemic embolism, *CHF* chronic heart failure, *AF* atrial fibrillation, *TyG* triglyceride-glucose, *TyG-BMI* triglyceride-glucose body mass index, *METS-IR* metabolic score for insulin resistance, *TG/HDL-C* triglyceride to high-density lipoprotein cholesterol ratio, *AUC* area under the receiver operating characteristic curve, *CI* confidence interval, *NRI* net reclassification improvement, *IDI* integrated discrimination improvement. Predictive performance was evaluated for 4-year IS/SE risk. “+” indicates that the corresponding IR surrogate index was added to the CHA₂DS₂-VASc score. *P* values for AUC, NRI, and IDI indicate comparisons with the CHA₂DS₂-VASc score model. Pairwise comparisons of AUC were performed among models incorporating different IR surrogate indices.
